# Targeted mutagenesis of *BnTT8* homologs controls yellow seed coat development for effective oil production in *Brassica napus* L.

**DOI:** 10.1111/pbi.13281

**Published:** 2019-11-11

**Authors:** Yungu Zhai, Kaidi Yu, Shengli Cai, Limin Hu, Olalekan Amoo, Lei Xu, Yang Yang, Boyuan Ma, Yangmiao Jiao, Chaofeng Zhang, Muhammad Hafeez Ullah Khan, Shahid Ullah Khan, Chuchuan Fan, Yongming Zhou

**Affiliations:** ^1^ National Key Laboratory of Crop Genetic Improvement Huazhong Agricultural University Wuhan China

**Keywords:** *Brassica napus*, seed coat colour, *TRANSPARENT TESTA 8*, CRISPR/Cas9, flavonoids, gene expression

## Abstract

Yellow seed is a desirable trait with great potential for improving seed quality in *Brassica* crops. Unfortunately, no natural or induced yellow seed germplasms have been found in *Brassica napus*, an important oil crop, which likely reflects its genome complexity and the difficulty of the simultaneous random mutagenesis of multiple gene copies with functional redundancy. Here, we demonstrate the first application of CRISPR/Cas9 for creating yellow‐seeded mutants in rapeseed. The targeted mutations of the *BnTT8* gene were stably transmitted to successive generations, and a range of homozygous mutants with loss‐of‐function alleles of the target genes were obtained for phenotyping. The yellow‐seeded phenotype could be recovered only in targeted mutants of both *BnTT8* functional copies, indicating that the redundant roles of *BnA09.TT8* and *BnC09.TT8b* are vital for seed colour. The *BnTT8* double mutants produced seeds with elevated seed oil and protein content and altered fatty acid (FA) composition without any serious defects in the yield‐related traits, making it a valuable resource for rapeseed breeding programmes. Chemical staining and histological analysis showed that the targeted mutations of *BnTT8* completely blocked the proanthocyanidin (PA)‐specific deposition in the seed coat. Further, transcriptomic profiling revealed that the targeted mutations of *BnTT8* resulted in the broad suppression of phenylpropanoid/flavonoid biosynthesis genes, which indicated a much more complex molecular mechanism underlying seed colour formation in rapeseed than in *Arabidopsis* and other *Brassica* species. In addition, gene expression analysis revealed the possible mechanism through which *BnTT8* altered the oil content and fatty acid composition in seeds.

## Introduction

Rapeseed (*Brassica napus* L., AACC, 2*n* = 38) is the third‐largest oilseed crop worldwide after soya bean and oil palm, accounting for approximately 16% of the entire global vegetable oil production (Hu *et al.*, 2017; Woodfield *et al.*, 2017). It provides not only edible oils for human diets and high‐quality animal feed proteins but also raw materials for industrial processes, such as biodiesel production. Achieving high oil yields, better oil and meal quality has always been the major breeding goals in rapeseed production. At present, most commercial rapeseed cultivars have brown to black seed colour. Previous studies showed that yellow‐seeded *B. napus* has a thinner seed coat, a reduced percentage of pigment and hull, and a greater content of oil and protein than the black‐seeded type (Marles and Gruber, 2004; Meng *et al.*, 1998; Tang *et al.*, 1997). With these superior characteristics, yellow seed is widely accepted as a good‐quality trait and is a focus of rapeseed research globally (Hong *et al.*, 2017; Jiang *et al.*, 2019; Lian *et al.*, 2017; Meng *et al.*, 1998; Qu *et al.*, 2013, 2016; Simbaya *et al.*, 1995; Tang *et al.*, 1997; Wen *et al.*, 2012).

As in *Arabidopsis*, the formation of seed colour is due to the deposition of the oxidized form of a flavonoid, proanthocyanidins (PAs, the so‐called condensed tannins), within the endothelial layer of the inner integument of the seed coat in *Brassica* species (Lepiniec *et al.*, 2006; Marles and Gruber, 2004). PA is synthesized through the common phenylpropanoid pathway into the flavonoid pathway to form anthocyanidin, and then PA is formed using anthocyanidin as a precursor (Debeaujon *et al.*, 2003). During seed maturation, colourless PA precursors, such as epicatechin, polymerize and oxidize to form dark brown PAs, altering the colour of the seed to dark brown or black (Lian *et al.*, 2017; Pourcel *et al.*, 2005). In *Arabidopsis*, the flavonoid pathway has been well characterized at the molecular level mainly by utilizing *transparent testa* (*tt*) and *tannin‐deficient seed* (*tds*) mutants, which influence flavonoid accumulation and modify seed coat pigmentation (Albert *et al.*, 2014; Lepiniec *et al.*, 2006; Shirley *et al.*, 1995; Xu *et al.*, 2014). They correspond to two groups of structural proteins, the so‐called early and late biosynthetic genes (EBGs and LBGs, respectively), transporters and regulatory factors. The EBG proteins catalyse the early biosynthetic steps and include chalcone synthase (CHS/TT4), chalcone isomerase (CHI/TT5), flavanone 3‐hydroxylase (F3H/TT6) and flavonoid 3′‐hydroxylase (F3’H/TT7), resulting in the formation of dihydroflavonols (the common precursors of flavonoids). The LBG proteins catalyse the late steps of the pathway and include the downstream enzymes dihydroflavonol‐4‐reductase (DFR/TT3), leucoanthocyanidin dioxygenase/anthocyanidin synthase (LDOX/ANS/TT18) and BANYULS/anthocyanidin reductase (BAN/ANR), in addition to the transporters glutathione S‐transferase 26 (GST26/GSTF12/TT19), auto‐inhibited H^+^‐ATPase isoform 10 (AHA10/TT13) and MATE transporter (TT12), as well as a laccase gene, *laccase 15* (*LAC15*/*TT10*), resulting in the production of anthocyanin/PAs (Lepiniec *et al.*, 2006; Xu *et al.*, 2014). This expression regulation of the genes involved in the flavonoid biosynthetic pathway is mainly controlled by different sets of transcription factors (TFs) in a tissue‐specific manner (Lepiniec *et al.*, 2006; Xu *et al.*, 2014, 2015). In *A. thaliana*, a ternary complex (known as the MBW complex) comprising three TF regulators, namely AtMYB123/TT2 (R2R3‐MYB), TT8 (basic helix‐loop‐helix, bHLH) and TTG1 (WD40 protein), plays a key role in activating PA‐specific genes in seed coat development (Xu *et al.*, 2014, 2015).

Accumulating evidence demonstrates that *TT8* is a central component of the well‐conserved complex that controls flavonoid accumulation in various crops (Escaray *et al.*, 2017; Li *et al.*, 2016; Li *et al.*, 2019; Lim *et al.*, 2017; Nemesio‐Gorriz *et al.*, 2017; Schaart *et al.*, 2013). Taking into account the close phylogenetic relationship between *Arabidopsis* and *Brassica*, *TT8* gene homologs could play comparable roles in *Brassica* species. Indeed, the yellow‐seeded trait of *B. rapa* var. yellow sarson is due to the loss of function of the *TT8* gene caused by an insertion of a transposable element in its intron (Li et al., 2012a,2012b). Similarly, natural mutations in two homologous *TT8* genes, *BjuA.TT8* and *BjuB.TT8*, control the yellow‐seeded trait in allotetraploid *B. juncea* (Padmaja *et al.*, 2014). In *B. napus*, studies have shown that most of the genes involved in the flavonoid biosynthetic pathway, including the homologs of *TT8*, are down‐regulated in yellow seed compared to black seed, indicating that they are evolutionarily conserved in rapeseed (Hong *et al.*, 2017; Jiang *et al.*, 2019; Qu *et al.*, 2013). However, the functions of these genes have yet to be verified. The *BnTT8* gene is therefore regarded as appropriate for manipulation in the breeding of yellow‐seeded varieties of rapeseed.

*Brassica napus* is an allotetraploid species that was formed by a recent hybridization of two diploid ancestors, *B. rapa* (AA) and *B. oleracea* (CC) (Chalhoub *et al.*, 2014). Although both of its diploid ancestors possess yellow seed genotypes with stable phenotypes and qualitative inheritance, *B. napus* naturally lacks yellow‐seeded mutants (Hong *et al.*, 2017; Jiang *et al.*, 2019; Lian *et al.*, 2017). It emerges mainly in rapeseed that is amphidiploid nature, having a minimum of two similar copies of most of its genes. Spontaneous and induced random mutagenesis usually induces single mutants of these copies, which in most cases do not have the preferred effect in rapeseed. Yellow‐seeded rapeseed has been exclusively developed through interspecific hybridization of *Brassica* species (*B. rapa*, *B. juncea*, *B. carinata*, and *B. oleracea*) or intergeneric hybridization with relevant genera (Rashid *et al.*, 1994; Meng *et al.*, 1998; Rahman et al., 2001; Wang *et al.*, 2005; Li *et al.*, 2009; Li et al., 2012a,2012b; Wen *et al.*, 2012). However, this strategy is time‐consuming and inefficient, as the introduced yellow seed trait exhibits extreme variation in seed colour stability, which is recognized as a major obstacle in yellow‐seeded rapeseed breeding (Liu *et al.*, 2005). Extensive studies in resynthesized rapeseed resources have also revealed the complexity of yellow seed trait introgression from related species, which made the yellow‐seeded feature in *B. napus* more difficult to study than *Arabidopsis* (Yu, 2013). Therefore, the utilization of new technologies that can simultaneously modify numerous copies of the gene of interest becomes imperative to create new genetic variation for seed coat colour in polyploid rapeseed.

In recent years, sequence‐specific nucleases (SSNs) have been demonstrated to be an amazing tool for the improvement of crops via site‐specific genome editing, and CRISPR/Cas9 is considered the most simple and efficient SSN. The CRISPR/Cas9 system has been effectively utilized in rapeseed to produce the targeted mutagenesis required to improve agronomic traits (Braatz *et al.*, 2017; Hu *et al.*, 2018; Li *et al.*, 2018; Yang *et al.*, 2017; Yang *et al.*, 2018; Zhai *et al.*, 2019; Zheng *et al.*, 2019).

Hence, we utilized the CRISPR/Cas9 system to generate efficient knockouts of *BnTT8* homeologs in the flavonoid biosynthesis pathway with stable transformation in rapeseed. In the T_1_ and T_2_ generations, mutant plants containing the desired gene modification but not the transferred DNA were obtained by segregation. This is the first report on the creation of yellow‐seeded mutants in rapeseed using CRISPR/Cas9 technology and provides valuable germplasm resources for further breeding of yellow‐seeded varieties of rapeseed. The current study also used transcriptomic analysis and metabolite profiling of mutant plants from *BnTT8* to investigate the molecular mechanism that regulates seed colour.

## Results

### Molecular cloning and characterization of *TT8* homologs in* B. napus*

Previous studies revealed that *TT8* gene function is essential for seed coat colour and is highly conserved in *Brassica* species. Loss‐of‐function *TT8* mutants showed yellow‐seeded traits in *B. rapa* and *B. juncea* (Li et al., 2012a,2012b; Padmaja *et al.*, 2014). Thus, *TT8* is one of the ideal candidates for creating yellow‐seeded germplasm resources in rapeseed. *B. napus* contains three *TT8* copies, one on chromosome A09 (*BnaA09g22810D*, designated as *BnA09.TT8*) and two tandem duplicates on chromosome C09 (*BnaC09g24860D*/*BnaC09g24870D*, designated *BnC09.TT8a*/*b*, respectively). To check for putative mutations in the target genes, we confirmed their genomic DNA sequences in the *B. napus* pure line J9707. In every one of the three cases, the sequenced ORFs matched the length of the high‐confidence gene models in the Darmor‐bzh reference genome assembly (Chalhoub *et al.*, 2014). As for *Arabidopsis* TT8, the predicted amino acid sequences of BnA09.TT8/BnC09.TT8b both contains several conserved domains: the N‐terminal MYB interaction region (MIR), the WD/AD and the bHLH domain in the C‐terminal region (Figs. S1 and S2). However, the predicted BnC09.TT8a lacked the MIR and WD/AD domain and part of the bHLH domain, which are essential for TT8 function (Figs. S1 and S2). Thus, the sequence analysis suggested that BnA09.TT8 and BnC09.TT8b encode functional bHLH proteins and that *BnC09.TT8a* is most likely a pseudogene. For this reason, *BnC09.TT8a* was not used in further experiments.

*BnA09.TT8* and *BnC09.TT8b* were 96.85% identical at the nucleotide level and share 97.89% amino acid identity, suggesting that these genes encode enzymes with similar functions. According to the sequence alignment of the two copies of *BnTT8* gene, polymorphisms distinguished the origins of these gene copies (Fig. S3).

Phylogenetic investigation showed that all *BnTT8* copies were clustered together with *AtTT8* and its homologs from different plant species in a well‐supported clade, all of which are involved in flavonoid biosynthesis (Fig. S4). It also revealed that *BnA09.TT8* and *BnC09.TT8a/b* were closely related to their homologs in *B. rapa* and *B. oleracea*, respectively (Fig. S4), which is in line with their origination from two diploid progenitors. Similarly, *B. oleracea* contained two tandem duplicates, that is, *BoTT8a* (*Bo9g086910*) and *BoTT8b* (*Bo9g086920*), with one truncated copy of *BoTT8a* (Figs. S1 and S2).

### Expression analysis of the *BnTT8* gene

**Figure 1:**
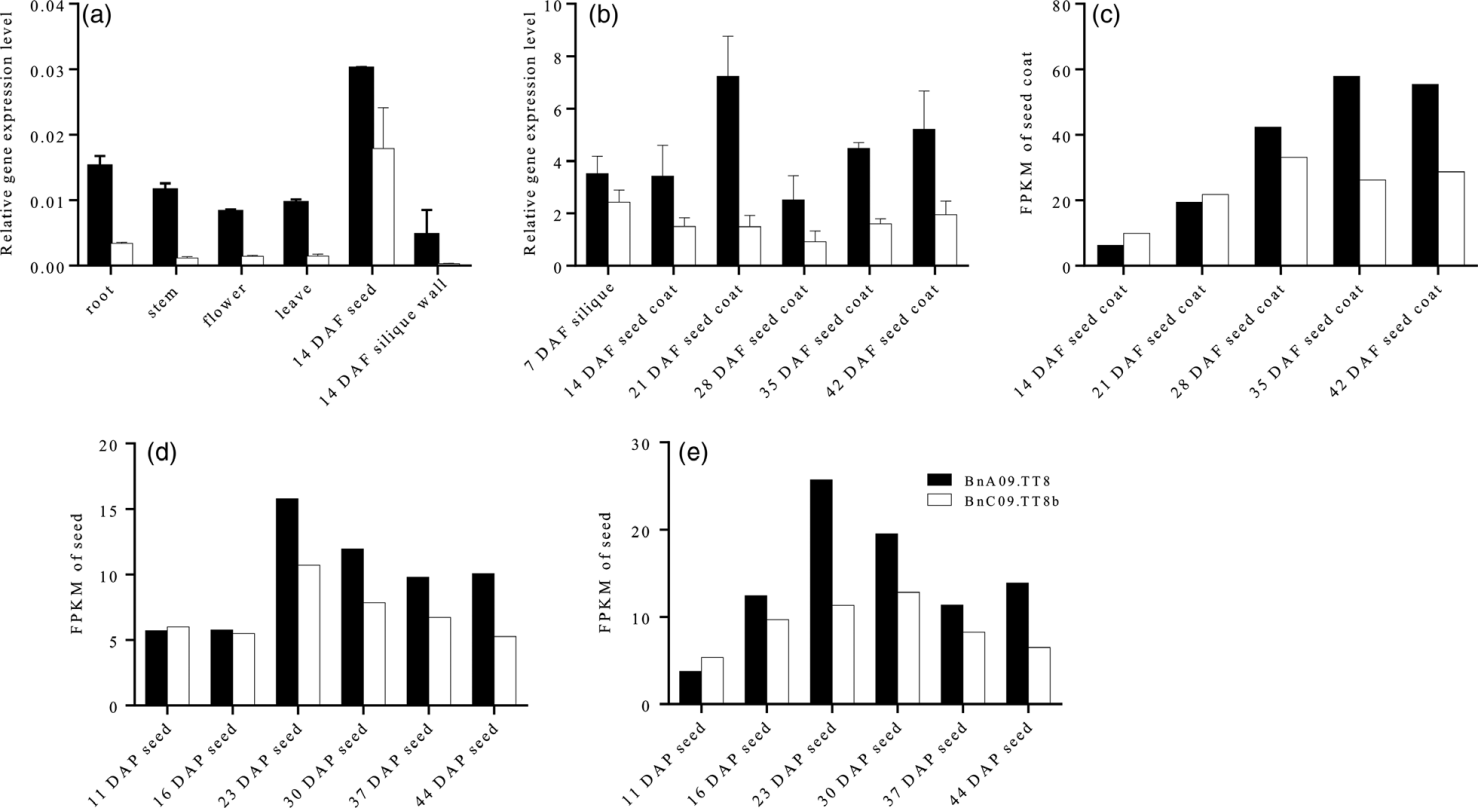
Expression pattern of *BnTT8* in rapeseed. Relative gene expression of *BnTT8* in various tissues of J9707 (a) and different stages of seed development in J9707 (b) were determined by qRT‐PCR; values are the means ± SE of three biological replicates. (c‐e) mRNA accumulation patterns for *BnTT8* were calculated by fragments per kilobase of transcript per million mapped reads (FPKM) based on public RNA‐seq data, including the seed coat transcriptomes of a brown‐seeded NIL (c; Hong et al., 2017), and the seed transcriptomes of two black‐seeded rapeseed inbred lines, 1L99 (d) and 1L363 (e), respectively (Shahid et al., 2019).

The expression pattern of *BnTT8* copies in J9707 was initially examined using quantitative real‐time PCR (qRT‐PCR) with RNA from different plant tissues (Fig. 1a). No transcript was identified for the *BnC09.TT8a*, but various amounts of transcript were detected for *BnA09.TT8* and *BnC09.TT8b* in all tissues, with the highest expression in seeds. Evidently, *BnA09.TT8* had a significantly higher expression level than *BnC09.TT8b* in these tissues. This result suggested that *BnTT8* principally plays a role in seed development.

To confirm and further characterize the expression of the *BnA09.TT8* and *BnC09.TT8b* copies, their expression was assessed during various stages of seed coat development: 7, 14, 21, 28, 35 and 42 days after flowering (DAF). The expression of both copies exhibited a steady increase from 7 DAF to a peak value at 21 DAF and then decreased at later stages (Fig. 1b). Overall, *BnA09.TT8* had a significantly higher expression level than *BnC09.TT8b* during seed formation. Analysis of mRNA accumulation patterns for *BnTT8* copies based on recent public RNA‐seq data in brown/black‐seeded rapeseed lines showed their expression profiles in the developing seeds with different genetic backgrounds (Fig. 1c–e). In all cases, the *BnC09.TT8a* transcripts were undetectable at all developing stages, whereas the expression of *BnA09.TT8* and *BnC09.TT8b* gradually increased during the early stage of seed development and peaked at 23 DAF (Fig. 1d and e) or 35 DAF (Fig. 1c). The expression level of *BnA09.TT8* was higher than that of *BnC09.TT8b* during later seed development, especially at and after the peaking stages (Fig. 1c–e). Thus, we further confirmed that the *B. napus* genome contains two functional *AtTT8* homologs, *BnA09.TT8* and *BnC09.TT8b*.

### Creation of CRISPR/Cas9‐targeted mutations in *BnTT8*

**Figure 2:**
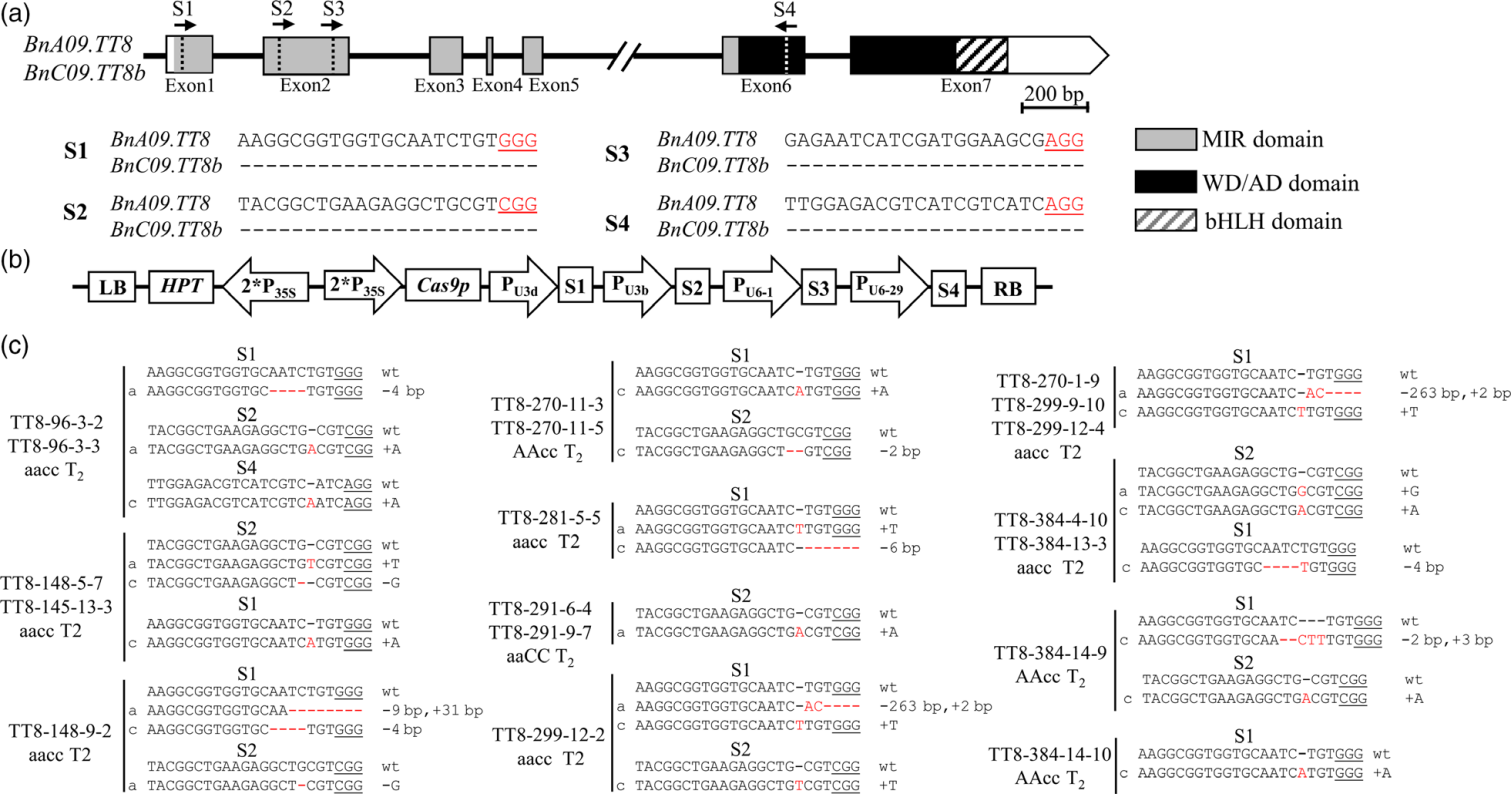
CRISPR/Cas9‐induced null mutants of *BnTT8* in *B. napus*. (a) The *BnTT8* gene model includes seven exons (box) separated by six introns (represented by the solid line). The positions of the N‐terminal MYB interaction region (MIR) domain, the WD/AD domain and the bHLH domain in the C‐terminal region are marked in the model. The vertical line in the gene model indicates the target site, and the arrow indicates the sgRNA direction. The target sequences are shown with the PAM underlined. (b) The CRISPR/Cas9 construct houses the following: a hygromycin resistance cassette consisting of the hygromycin phosphotransferase coding sequence driven by the cauliflower mosaic virus 35S promoter; a Cas9 expression cassette comprising the sequence encoding Cas9 driven by P_35S_; and four sgRNAs (S1‐S4) driven by the U3d, U3b, U6‐1 and U6‐29 promoters from *Arabidopsis*. (c) Sequences at the sgRNA target sites of *BnTT8* homozygous mutants in T_2_ generation. The protospacer adjacent motif (PAM) is underlined, and nucleotide indels are marked in red, with details labelled at right, ‘a’ and ‘c’ represent the mutated alleles of the target gene on *BnA09.TT8* and *BnC09.TT8b*, respectively. ‘aaCC’, ‘AAcc’ and ‘aacc’ represent homozygous mutations of the target gene in *BnA09.TT8*, *BnC09.TT8b* and both copies, respectively.

To generate Cas9‐induced knockout mutations in the functional copies of *BnTT8*, four sgRNAs were designed using the CRISPR‐P program (Lei *et al.*, 2014; Fig. 2a). Three of these sgRNAs, that is, sgRNA1 (S1) through sgRNA3 (S3), were designed to target the MIR domain, and the targeting sequence of sgRNA4 (S4) was within the WD/AD domain; this was expected to induce mutations in the functional domain of the gene and thus inactivate the BnTT8 protein (Figs 2a and S3). The sgRNAs matched well with *BnA09.TT8* and *BnC09.TT8b* (Fig. 2a). A CRISPR/Cas9 construct containing these four sgRNAs with Cas9 driven by the P_35S_ promoter (Fig. 2b) was produced based on the CRISPR/Cas9 multiplex genome‐editing vector as previously described by Yang *et al. *(2018). The resulting construct was transformed into J9707 using *Agrobacterium*‐mediated transformation, and 333 T_0_‐positive transgenic plants were generated. A total of 48 targeted mutants were identified by Sanger DNA sequencing of the PCR products of the target sites, with five plants showing a visible knockout phenotype (i.e., yellow seed; Table 1).

**Table 1:** Genotypic analysis of *BnTT8* mutants and their transmission to T_1_ and T_2_ generations

Plant ID	Generation	Positive	Genotype at targets of *BnA09.TT8*				Genotype at targets of *BnC09.TT8b*				Seed colour
			S1	S2	S3	S4	S1	S2	S3	S4	
TT8‐96	T0	Y	Hetero	Homo (+1bp)	wt	Hetero	Hetero	/	wt	Homo (+1bp)	Yellow
TT8‐96‐2	T1	Y	Homo (+1bp)	Homo (+1bp)	wt	Hetero	Hetero	/	wt	Homo (+1bp)	Yellow
TT8‐96‐3	T1	N	Homo (−4bp)	Homo (+1bp)	wt	wt	Hetero	/	wt	Homo (+1bp)	Yellow
TT8‐96‐3‐2	T2	N	Homo (−4bp)	Homo (+1bp)	wt	/	/	/	/	Homo (+1bp)	Yellow
TT8‐96‐3‐3	T2	N	Homo (−4bp)	Homo (+1bp)	wt	wt	/	/	wt	Homo (+1bp)	Yellow
TT8‐148	T0	Y	Hetero	Homo (−1bp)	wt	Hetero	Homo (−4bp)	/	wt	wt	Yellow
TT8‐148‐5	T1	Y	Homo (+31bp, −9bp)	Homo (−1bp)	wt	Homo (+1bp)	Homo (−4bp)	wt	wt	wt	Yellow
TT8‐148‐5‐7	T2	Y	Homo (+31bp,‐9bp)	Homo (−1bp)	wt	/	Homo (−4bp)	wt	wt	/	Yellow
TT8‐148‐9	T1	Y	Homo (+31bp, −9bp)	Homo (−1bp)	wt	Homo (+1bp)	Homo (−4bp)	wt	wt	wt	Yellow
TT8‐148‐9‐2	T2	Y	Homo (+31bp, −9bp)	Homo (−1bp)	wt	/	Homo (−4bp)	wt	wt	/	Yellow
TT8‐145	T0	Y	Hetero	Homo (+1bp)	wt	Hetero	Homo (+1bp)	/	wt	wt	Yellow
TT8‐145‐3	T1	Y	wt	Homo (+1bp)	wt	Hetero	Homo (+1bp)	wt	wt	wt	Yellow
TT8‐145‐13‐3	T2	Y	wt	Homo (+1bp)	wt	/	Homo (+1bp)	Homo (−1bp)	wt	/	Yellow
TT8‐281	T0	Y	Homo (+1bp)	/	/	Hetero	Homo (−6bp)	/	/	Hetero	Yellow
TT8‐281‐5	T1	Y	Homo (+1bp)	wt	wt	wt	Homo (−6bp)	wt	wt	wt	Yellow
TT8‐281‐5‐5	T2	Y	Homo (+1bp)	wt	wt	wt	Homo (−6bp)	wt	wt	wt	Yellow
TT8‐299	T0	Y	Homo (−263bp, +2bp)		wt	Hetero	Homo (+1bp)	Hetero	/	wt	Yellow
TT8‐299‐9	T1	Y	Homo (−263bp, +2bp)		/	Hetero	Homo (+1bp)	Hetero	wt	wt	Yellow
TT8‐299‐9‐10	T2	N	Homo (−263bp, +2bp)		wt	/	Homo (+1bp)	Hetero	/	/	Yellow
TT8‐299‐12	T1	Y	Homo (−263bp, +2bp)		wt	Homo (−3bp)	Homo (+1bp)	Hetero	wt	wt	Yellow
TT8‐299‐12‐2	T2	N	Homo (−263bp, +2bp)		wt	/	Homo (+1bp)	Homo (+1bp)	wt	wt	Yellow
TT8‐299‐12‐4	T2	Y	Homo (−263bp, +2bp)		wt	/	Homo (+1bp)	wt	wt	/	Yellow
TT8‐291	T0	Y	wt	Hetero	/	wt	Wt	wt	wt	wt	Black
TT8‐291‐6	T1	Y	wt	Homo (+1bp)	wt	wt	wt	wt	wt	wt	Black
TT8‐291‐6‐4	T2	Y	wt	Homo (+1bp)	wt	/	wt	wt	wt	wt	Black
TT8‐291‐9	T1	N	wt	Hetero	wt	wt	wt	wt	wt	wt	Black
TT8‐291‐9‐7	T2	N	wt	Homo (+1bp)	wt	/	wt	wt	wt	wt	Black
TT8‐384	T0	Y	wt	Hetero	/	wt	Hetero	Homo (+1bp)	wt	wt	Black
TT8‐384‐4	T1	Y	wt	Homo (+1bp)	wt	wt	Homo (−4bp)	Homo (+1bp)	wt	wt	Yellow
TT8‐384‐4‐10	T2	Y	wt	Homo (+1bp)	wt	/	Homo (−4bp)	Homo (+1bp)	wt	/	Yellow
TT8‐384‐13‐3	T2	Y	wt	Homo (+1bp)	wt	/	Homo (−4bp)	Homo (+1bp)	wt	/	Yellow
TT8‐384‐14	T1	N	wt	wt	wt	wt	Hetero	Homo (+1bp)	wt	wt	Black
TT8‐384‐14‐9	T2	Y	wt	wt	wt	wt	Homo (−2bp, +3bp)	Homo (+1bp)	wt	/	Black
TT8‐384‐14‐10	T2	Y	wt	wt	wt	wt	Hetero	Homo (+1bp)	wt	/	Black
TT8‐270	T0	Y	wt	wt	wt	wt	biallelic	/	/	wt	Black
TT8‐270‐1	T1	Y	wt	wt	wt	wt	Hetero	/	/	wt	Black
TT8‐270‐1‐9	T2	N	Homo (−263bp,+2bp)		wt	wt	Homo (+1bp)	Hetero	/	wt	Yellow
TT8‐270‐11	T1	N	wt	wt	wt	wt	Hetero	/	wt	wt	Black
TT8‐270‐11‐3	T2	N	wt	wt	wt	wt	Homo (+1bp)	Homo (−2bp)	wt	wt	Black
TT8‐270‐11‐5	T2	N	wt	wt	wt	wt	Homo (+1bp)	Homo (−2bp)	wt	wt	Black

Hetero, heterozygous; Homo, homozygous; ‘−’ and ‘+’ indicate the deletion and insertion of the indicated number of nucleotides.

**Figure 3:**
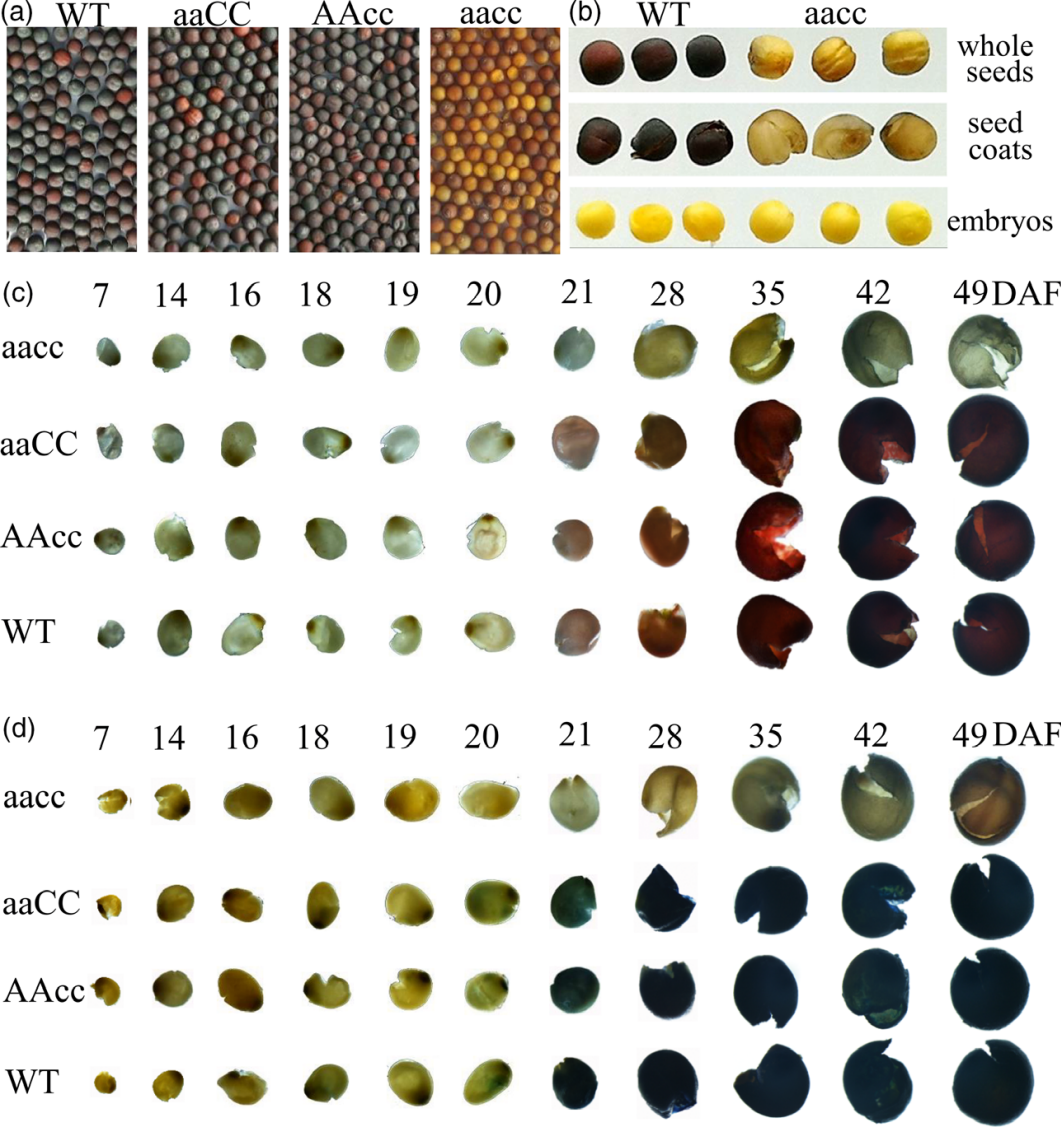
Seed colour phenotypes of the *BnTT8* mutants. (a) Mature seeds from WT and *BnTT8* mutants. ‘aaCC’, ‘AAcc’ and ‘aacc’ represent homozygous mutations of the target gene in *BnA09.TT8*, *BnC09.TT8b* and both copies, respectively. (b) Dissecting seed coat and embryo of WT and *BnTT8* double mutants. (c‐d) Seed coats from WT and *BnTT8* mutants after vanillin (c) and DMACA (d) staining. DAF, days after flowering.

To acquire stable lines with targeted mutations, eight independent T_0_ editing lines of *BnTT8* were self‐pollinated to produce T_1_ and T_2_ progeny. The targeted mutations of progeny from these T_0_ lines were verified by high‐throughput tracking of mutations (Hi‐TOM) sequencing analysis of the target sites. A total of eighteen T_2_ lines with homozygous mutations in *BnTT8* were detected, including two *BnA09.TT8* single mutants, four *BnC09.TT8b* single mutants and twelve *BnTT8* double mutants (Table 1; Fig. 2c). All of these detected homozygous mutations at the target sites within *BnTT8* were predicted to cause frame shifts and result in non‐functional proteins, with the exception of T_2_ line TT8‐281‐5‐5, which had a 6‐nucleotide‐long deletion resulting in two amino acid (Val31 and Gly32) deletions in the conserved MIR domain of *BnC09.TT8b* (Table 1; Fig. 2c; Fig. S5). As expected, all of those double mutants could produce yellow seeds, while the single mutants showed a comparable phenotype to that of the WT (Table 1; Fig. 3a). Thus, both copies of the *BnTT8* gene function redundantly in yellow seed development.

We performed PCR assays of the T_1_ and T_2_ plants to explore the potential for targeted changes without integrating foreign DNA into the rapeseed genome. A variety of *BnTT8* homozygous T‐DNA‐free mutants were identified (Table 1).

### Targeted mutations in *BnTT8* lead to defective PA accumulation in the inner seed coat

Dissecting the seed coat and embryo of WT and *BnTT8* double‐mutant seeds clearly shows that the seed colour difference is mainly determined by the seed coat, not by the embryo (Fig. 3b). The brown to dark colour of mature seeds in *Brassica* species is due to the PA oxidations during seed desiccation that cause the accumulation of colourless compounds in the seed coat (Lepiniec *et al.*, 2006; Marles and Gruber, 2004). The dissected seed coats of WT, single (TT8‐291‐6‐4, TT8‐291‐9‐7, TT8‐384‐14‐9, TT8‐270‐11‐3) and double (TT8‐96‐3‐2, TT8‐148‐5‐7) homozygous mutants of *BnTT8* were used for the vanillin and DMACA staining test to determine the dynamic variation of PA accumulation during seed development. Red coloration with vanillin and blue coloration with DMACA started at 21 DAF in the black seed coats of both WT and single mutants, and the colour gradually became darker during development (Fig. 3c and d). The double‐mutant seeds at any stage were not obviously stained (Fig. 3c and d).

**Figure 4:**
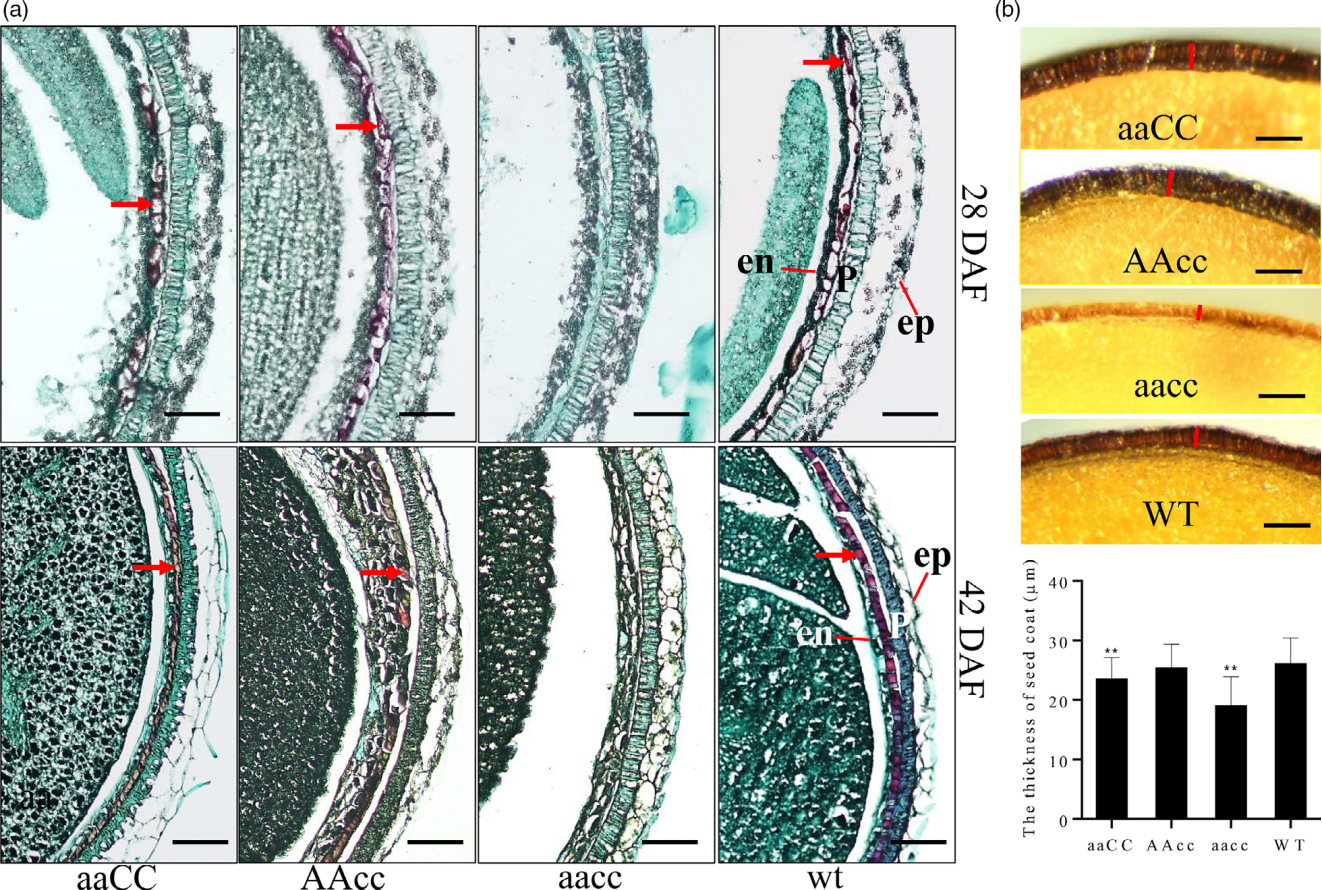
Mutations in the *BnTT8* gene affected seed development and blocked the PA deposition in the seed coat. (a) Microscopy of Safranin O and Fast Green‐stained transverse sections from 28 DAF and 42 DAF seeds of WT and *BnTT8* mutants. ‘aaCC’, ‘AAcc’ and ‘aacc’ represent homozygous mutations of the target gene in *BnA09.TT8*, *BnC09.TT8b* and both copies, respectively. Arrowheads indicate the red‐stained PA deposited in the endothelial cell of seed coats; ep, epidermis; p, palisade layer; en, endothelial cell layer. Bars, 100 µm. (b) Microscopic observation of the thickness of mature seed coat. Bars, 50 µm. Data are presented as means ± SE (*n* ≥ 15); Student’s *t*‐test was used for statistical analysis between the mutant and its WT (**, *P* < 0.01).

Microscopy of Safranin O and Fast Green‐stained transverse sections showed that the accumulated PAs were deposited in the endothelial cells of the WT and single‐mutant seed coats (indicated by arrowheads in Fig. 4a). However, the red‐stained PA was completely absent in the double‐mutant seed coat (Fig. 4a). Additionally, the seed coat thickness had the most drastic reduction in the double mutant, by 27.0% lower relative to WT seeds; the *BnA09.TT8* single mutant showed a significant decrease (9.8%) in the thickness of the seed coat with respect to WT plants, whereas this trait in the *BnC09.TT8b* single mutant was similar to the WT trait (Fig. 4b).

Together, these findings indicate that the disruption of *BnTT8* affected seed development and hindered the PA deposition in the inner layer of the seed coat, which is consistent with previously described *tt8* mutant seed phenotypes in *Arabidopsis* and other *Brassica* species (Li et al., 2012a,2012b; Nesi *et al.*, 2000; Padmaja *et al.*, 2014). The findings also suggest that the most important stage of seed coat colour formation occurs at 21 DAF in *B. napus*.

### Effects of *BnTT8* targeted mutants on seed oil and protein contents, fatty acid (FA) composition and yield‐related traits

To characterize the effect of the *BnTT8* targeted mutations on oil and protein contents and FA composition, all double homozygous T_0_ and T_2_ lines with diverse frame‐shift targeted mutations (Table 1) were chosen for subsequent phenotypic characterization. The oil content from WT seeds was 45.34%–47.32% dry weight (Table 2). However, the oil contents of the double‐mutant seeds were approximately 51.80% in T_0_ plants and 48.01% in T_2_ plants, increased by 9.47% and 5.89% relative to WT seeds, respectively (Table 2). The protein contents of the double‐mutant seeds were also simultaneously significantly increased by 16.95% in T_0_ plants and 16.00% in T_2_ plants relative to WT seeds, respectively (Table 2), which is thought to improve the nutritional quality of the oilseed. Examination of FA profiles in T_0_ and T_1_ plants showed a consistent alteration in the FA composition in *BnTT8* mutants, including increases in palmitic acid (C16:0), linoleic acid (C18:2) and linolenic acid (C18:3) and decreases in stearic acid (C18:0) and oleic acid (C18:1) relative to WT seeds (Table 2).

**Table 2:** Seed oil and protein contents, and FA composition of *BnTT8* double mutant (aacc) and WT in T_0_ and T_2_ lines

Genotype	Generation	Oil content (%)	Protein content (%)	C16:0 (%)	C18:0 (%)	C18:1 (%)	C18:2 (%)	C18:3 (%)	C20:1 (%)
WT	T0	47.32 ± 0.58	15.40 ± 0.28	3.92 ± 0.04	2.57 ± 0.13	72.22 ± 0.66	13.84 ± 0.25	6.85 ± 0.36	0.60 ± 0.10
aacc	T0	51.80 ± 0.74**	18.01 ± 1.13**	4.17 ± 0.08**	1.65 ± 0.21**	64.45 ± 0.74**	19.69 ± 0.38**	9.26 ± 0.80**	0.77 ± 0.03**
WT	T2	45.34 ± 1.12	18.94 ± 2.65	3.95 ± 0.24	2.83 ± 0.65	68.13 ± 4.23	16.78 ± 3.28	7.46 ± 1.47	0.86 ± 0.11
aacc	T2	48.01 ± 1.08**	21.97 ± 1.64**	4.41 ± 0.43**	2.17 ± 0.47**	63.84 ± 2.63**	19.84 ± 2.32**	8.92 ± 0.90**	0.83 ± 0.10

The data represent the mean ± SD; Student’s *t*‐test was used for statistical analysis between the mutant and its corresponding WT (*, *P* ≤ 0.05; **, *P* ≤ 0.01).

To comprehensively characterize the *BnTT8* mutant phenotypes, single (TT8‐291‐9‐7, TT8‐270‐11‐3, TT8‐384‐14‐9, TT8‐384‐14‐10) and double (TT8‐299‐12‐2, TT8‐270‐1‐9, TT8‐384‐13‐3, TT8‐96‐3‐2, TT8‐281‐5‐5) homozygous mutant T_3_ lines without T‐DNA were grown in the field following a randomized block design with three replicates. The oil content in the single‐mutant seeds of *BnTT8* was similar to that in the WT control ranging from 44.94% to 45.50% (Table 3). However, the oil contents of all double‐mutant lines, which ranged from 46.69% to 48.97%, were significantly increased (by 5.90% on average) relative to WT seeds (Table 3). The protein content of the single‐mutant seeds was similar to that of WT, whereas this trait in all double‐mutant lines was higher than that in the WT control with two of these lines showing significant differences (Table 3). The overall alteration in FA composition in double mutants showed a similar trend to the T_0_ and T_1_ generations, with some variations among different lines (Table 3). We also evaluated the yield‐related traits of these T_3_ mutant lines. Although different degrees of changes in these yield‐related traits were observed among different lines, the seed yields of these mutants were similar to those of the WT (Table S2). Thus, the simultaneous targeted mutation of *BnA09.TT8* and *BnC09.TT8b* conferred a high oil yield potential with modified FA composition and improved the nutritional quality. Therefore, the *BnTT8* double mutants generated in this study could serve as excellent starting materials for rapeseed breeding.

**Table 3:** Seed oil and protein contents, and FA composition of WT, single and double homozygous mutant of *BnTT8* without T‐DNA in T_3_ generation

Materials	Genotype	Oil content (%)	Protein content (%)	C16:0 (%)	C18:0 (%)	C18:1 (%)	C18:2 (%)	C18:3 (%)	C20:1 (%)
J9707	WT	45.40 ± 0.36	19.06 ± 0.43	3.99 ± 0.13	2.96 ± 0.41	67.54 ± 2.16	17.20 ± 1.43	7.54 ± 0.62	0.78 ± 0.05
TT8‐291‐9‐7	aaCC	45.22 ± 1.04	18.22 ± 0.63	4.10 ± 0.15	3.23 ± 0.27	69.98 ± 0.48*	14.39 ± 0.36**	7.61 ± 0.09	0.69 ± 0.02**
TT8‐270‐11‐3	AAcc	44.57 ± 0.52	20.09 ± 0.60	4.25 ± 0.10**	2.92 ± 0.07	68.53 ± 0.52	16.46 ± 0.48	7.18 ± 0.14	0.66 ± 0.01**
TT8‐384‐14‐9	AAcc	45.19 ± 0.09	19.19 ± 0.07	4.43 ± 0.11**	3.72 ± 0.07**	68.39 ± 0.49	16.19 ± 0.37	6.59 ± 0.11*	0.67 ± 0.01**
TT8‐384‐14‐10	AAcc	44.94 ± 0.00	19.19 ± 0.08	4.04 ± 0.10	2.57 ± 0.05	68.87 ± 0.53	16.83 ± 0.32	6.88 ± 0.30	0.81 ± 0.01
TT8‐299‐12‐2	aacc	47.92 ± 1.09**	20.15 ± 0.58*	4.02 ± 0.05	2.22 ± 0.06**	62.43 ± 0.31**	21.34 ± 0.23**	9.22 ± 0.10**	0.77 ± 0.01
TT8‐270‐1‐9	aacc	48.66 ± 0.74**	20.21 ± 0.24*	4.20 ± 0.01*	2.53 ± 0.11	63.41 ± 0.28**	21.29 ± 0.27**	7.80 ± 0.10	0.77 ± 0.01
TT8‐384‐13‐3	aacc	48.97 ± 0.95**	19.43 ± 0.36	4.99 ± 0.13**	2.63 ± 0.09	62.77 ± 0.13**	20.87 ± 0.29**	8.02 ± 0.10	0.71 ± 0.02*
TT8‐96‐3‐2	aacc	46.69 ± 0.56*	19.38 ± 0.81	4.71 ± 0.02**	2.86 ± 0.09	61.48 ± 0.06**	23.25 ± 0.16**	7.01 ± 0.01	0.68 ± 0.01**
TT8‐281‐5‐5	aacc	48.16 ± 0.80**	19.12 ± 0.10	4.49 ± 0.12**	2.80 ± 0.06	63.69 ± 0.88**	20.41 ± 0.56**	7.87 ± 0.18	0.73 ± 0.01

The data represent the mean ± SD; Student’s *t*‐test was used for statistical analysis between the mutant and its corresponding WT (*, *P* < 0.05; **, *P* < 0.01).

### Off‐target activity of CRISPR/Cas9 in T_0_ transgenic *B. napus* plants

To ascertain whether off‐targeting occurred in the present study, we searched the *B. napus* genome for putative off‐target sites with high homology to the three sgRNAs that detected on‐target mutations according to the CRISPR‐P program (Lei *et al.*, 2014). These potential off‐target sites are listed in Table S3. There were 8, 9 and 9 putative off‐target sites for S1, S3 and S4, respectively (Table S3).

High‐throughput sequencing of the PCR products of these 26 potential sites from 30 T_0_ gene‐edited plants exhibited no mutations (Table S3), indicating that the off‐target effect is negligible when the sgRNA specificity is well considered according to the genome sequence. Thus, the CRISPR/Cas9 system has a high specificity for targeted mutagenesis in *B. napus*.

### *BnTT8* regulates the expression of the phenylpropanoid and flavonoid biosynthesis genes

As the investigation above showed that the PAs accumulated more accumulated after 21 DAF in the seed coat of WT (Fig. 3c and d), we collected the developing seed coats at 14 DAF and 35 DAF for comparison of the expression profiles between the *BnTT8* double mutants (TT8‐299‐12‐2, TT8‐96‐3‐2, TT8‐270‐1‐9) and corresponding WT to investigate the transcriptional changes underlying the seed coat colour phenotypes (Table S4). A total of 52 962 genes were expressed in the developing seed coats at the two stages and were included in the subsequent analysis. The Pearson correlation coefficient between any two of the three biological replicates at each stage was high (*R* = 0.91–0.98) in both mutant and WT, indicating that the sequencing data utilized in the present study were highly reliable (Fig. S6).

Comparison of transcript abundances in these developing seed coats uncovered 1298 differentially expressed genes (DEGs) between each double mutant and its corresponding WT, among which 145 DEGs were shared in common by the two stages (Table S5; Fig. S7). The number of DEGs at stage 14 DAF (963 DEGs) was almost twofold of that at stage 35 DAF (480 DEGs), indicating extensive changes in gene expression preceding observable changes in seed coat colour (Fig. S7). Generally, 732 genes exhibited up‐regulation, and 711 genes exhibited down‐regulation in the mutant seed coat, which might be associated with seed colour development (Fig. S8). Gene Ontology (GO) and Kyoto Encyclopedia of Genes and Genomes (KEGG) enrichment analysis for these identified DEGs showed that the phenylpropanoid and flavonoid metabolic processes were significantly enriched among down‐regulated DEGs in mutants relative to WT, especially at 35 DAF, an important stage during the formation of seed coat colour (Figs. S9‐S11; Tables S6‐S8).

**Figure 5:**
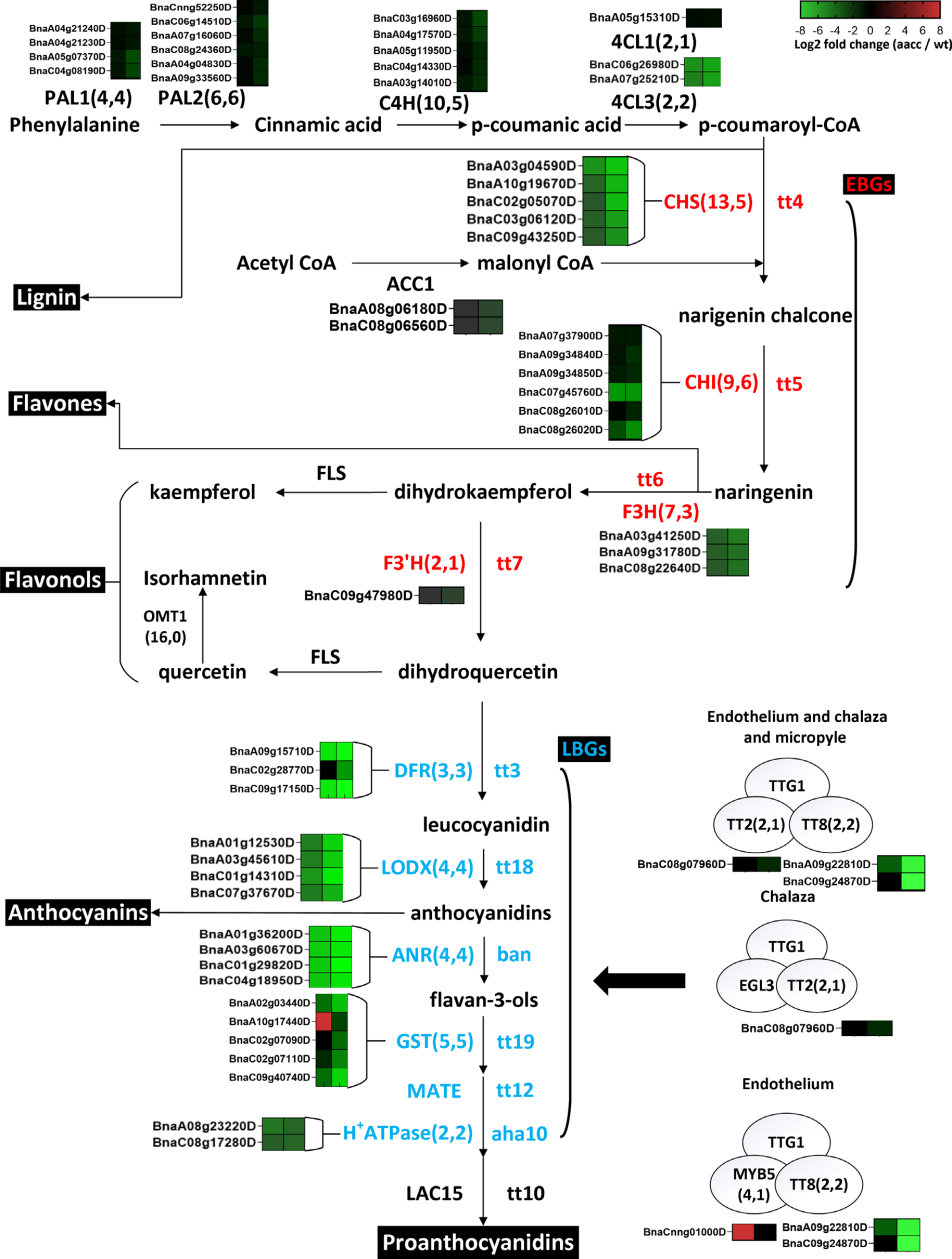
Phenylpropanoid and flavonoid biosynthetic genes regulated by *BnTT8* in rapeseed. The gene copy numbers from the genome and from the DEGs in the present study are listed in parentheses, respectively. Log_2_ fold changes (aacc/WT) of DEGs at 14 DAF and 35 DAF are illustrated with green (down‐regulation) or red (up‐regulation) boxes ordered left to right. Names of genes are indicated in capital letters, and corresponding mutants in lower‐case italics. EBGs and LBGs are indicated in red and blue, respectively. Abbreviations: PAL, phenylalanine ammonia‐lyase; C4H, cinnamic acid 4‐hydroxylase; 4CL, 4‐coumarate:coenzyme A ligase; CHS, chalcone synthase; CHI, chalcone isomerase; F3H, flavanone 3‐hydroxylase; F3’H, flavonoid 3’‐hydroxylase; DFR, dihydroflavonol‐4‐reductase; LDOX, leucoanthocyanidin dioxygenase/anthocyanidin synthase; ANR, anthocyanidin reductase; GST26, glutathione S‐transferase 26; MATA*,* multidrug and toxic efflux transporter; AHA10, auto‐inhibited H^+^‐ATPase isoform 10; LAC15, laccase 15; EGL3, enhancer of glabra 3; TT2/8, transparent testa 2, 8; TTG1, transparent testa glabra 1; EBGs, early biosynthetic genes; LBGs, late biosynthetic genes.

In the general phenylpropanoid pathway, the expression of genes encoding three classes of enzymes, namely PAL, C4H and 4CL, was suppressed to different extents at least in the 35 DAF mutant seed coat, including four copies of *BnPAL1*, six copies of *BnPAL2*, five copies of *BnC4H*, two copies of *Bn4CL3* and a copy of *Bn4CL1* (Fig. 5; Table S8).

In flavonoid biosynthesis, all the structural genes showed notable down‐regulation at both stages in most cases, including five copies of *BnCHS*, six copies of *BnCHI*, three copies of *BnF3H* and *BnDFR*, two copies of *BnCHIL*, a copy of *BnF3’H*, and four copies of *BnLODX* and *BnBAN* genes (Fig. 5; Table S8). Similarly, three key transporter genes that act downstream of the structural genes were also repressed in the mutant seed coat, including four copies of *BnGST26* and two copies of *BnTT12* and *BnAHA10* (Fig. 5; Table S8). A homolog of *LAC15*, encoding a laccase‐like oxidase involved in the formation of flavonoid end products, was down‐regulated more than sixfold in the mutants compared with WT (Fig. 5; Table S8). The expression of several key regulatory genes controlling PA accumulation in the seed coat was also changed at different stages in the mutants (Fig. 5; Table S8). As the targeted mutated gene, *BnA09.TT8* was down‐regulated by almost fivefold at 14 DAF and 330‐fold at 35 DAF, and *BnC09.TT8b* was only down‐regulated at 35 DAF (Fig. 5; Table S8), which indicates that *BnTT8* controls its own transcription in the seed coat similar to that in *Arabidopsis* (Baudry *et al.*, 2006). Again, no transcript was detected for *BnC09.TT8a*, which further confirmed it as a non‐functional copy. The down‐regulated DEGs also included two copies of *BnMYBL2* and a copy of *BnTT2* (Fig. 5; Table S8). Conversely, a copy of *BnMYB5* as a key member of the MBW ternary complexes showed significant up‐regulation only at 14 DAF. For flavone biosynthesis, one out of 16 copies of *BnFOMT* was markedly down‐regulated at both stages (Table S10).

To validate the RNA‐seq data, subsets of 29 DEGs in developing seed coats were chosen for qRT‐PCR analysis. These genes included 16 DEGs involved in phenylpropanoid and flavonoid biosynthesis and 13 randomly chosen DEGs. Linear regression analysis showed very high correlation coefficients (*R* = 0.803–0.900; Fig. S12) between the transcript levels assayed by the two analytic systems, further confirming the reliability of the RNA‐seq data.

Together, these results agree with the mutation phenotypes of *BnTT8* and further demonstrate that *BnTT8* is a key regulator during flavonoid biosynthesis in the seed coat of *B. napus*.

### Targeted mutations in *BnTT8* change flavonoid composition in the seeds

**Figure 6:**
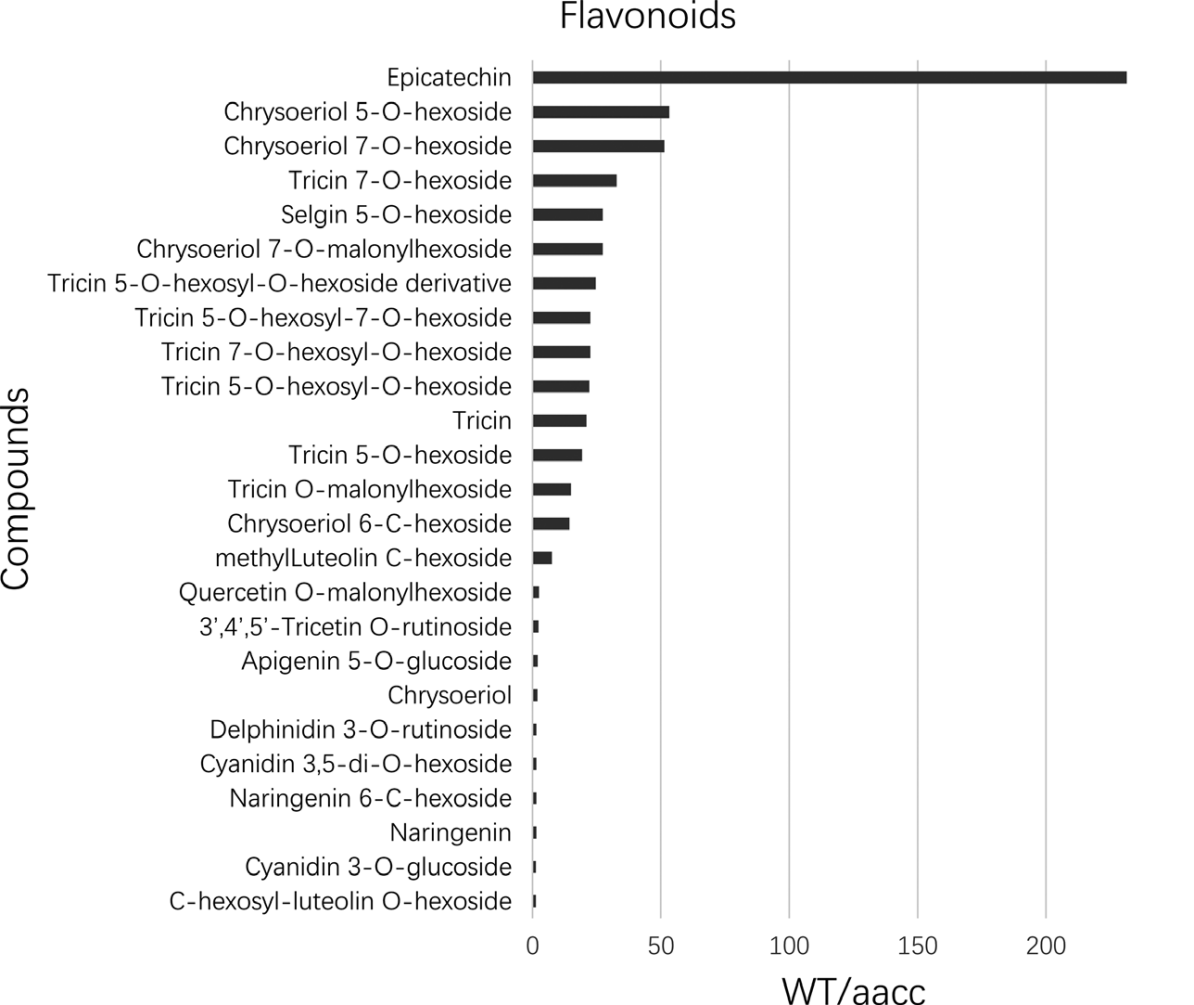
The identified flavonoid metabolites in mature seeds with significant difference between WT and the *BnTT8* double mutant (aacc). The level of all of these metabolites decreased in mutant seeds. And they are listed in the order of decreased fold changes (WT/aacc) from top to down.

To assess the impact of targeted mutation of *BnTT8* on the flavonoid metabolic pathway, metabolite profiling of the double‐mutant (TT8‐299‐12‐2, TT8‐96‐3‐2, TT8‐270‐1‐9) and its WT control seeds was analysed using an LC‐ESI‐MS/MS system. Approximately half of the identified flavonoid metabolites showed a significant difference between the double‐mutant and WT seeds (Tables S9, S10). All of these differential metabolites were lower in mutant seeds than in WT seeds (Table S10). Epicatechin, the PA precursor, showed the most drastic reduction in the mutants, by more than 230‐fold lower relative to WT seeds (Table S10; Fig. 6). Targeted mutation of *BnTT8* also blocked the accumulation of other flavonoid compounds, including flavones, anthocyanidins and naringenin (Table S10; Fig. 6). Interestingly, the flavone compounds were obviously enriched in the differential metabolite profiling (Table S10; Fig. 6). These results are in line with the phenotypes and the transcriptomic analysis of the mutant seeds and further demonstrate that *BnTT8* plays an important role in the regulatory network controlling flavonoid accumulation during seed development in *B. napus*.

### The *BnTT8* targeted mutation results in altered expression of genes involved in FA biosynthesis during seed development

**Figure 7:**
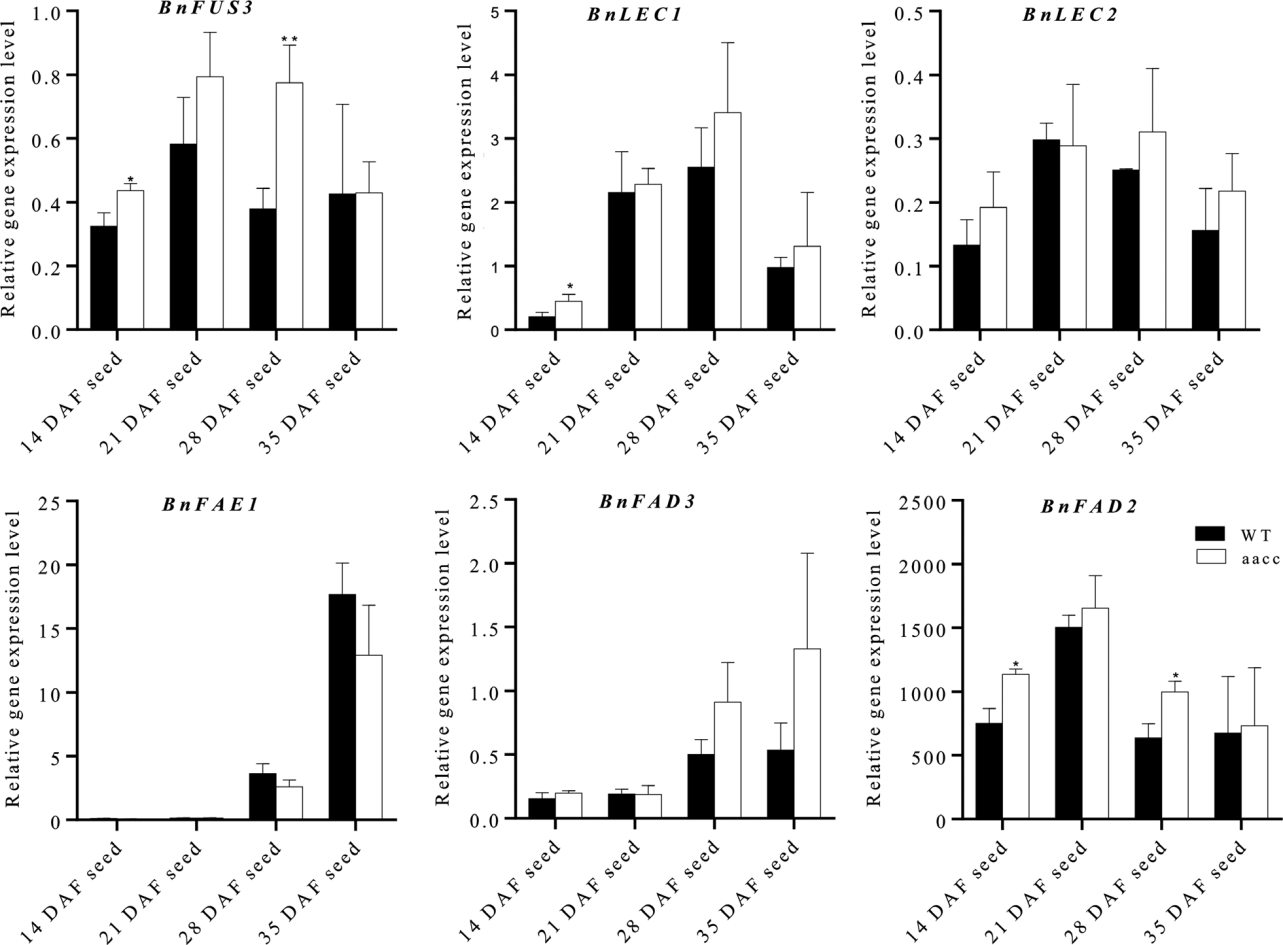
The *BnTT8* targeted mutation resulted in altered expression of genes involved in FA biosynthesis during seed development. Relative gene expression in different stages of seeds of WT and the *BnTT8* double mutant (aacc) was determined by qRT‐PCR with normalization to *BnUBC10*. Values are the means ± SE of three biological replicates. Student’s *t*‐test was used for statistical analysis between the mutant and its WT (*, *P* < 0.05).

Since targeted mutation of *BnTT8* resulted in significantly increased oil content and alteration of FA composition, we speculated that *BnTT8* participates in the regulation of genes involved in the FA biosynthesis pathway during seed development. To test this hypothesis, the expression of several critical TFs controlling seed development and FA accumulation, such as *LEAFY COTYLEDON1* (*LEC1*), *LEC2* and *FUSCA3* (*FUS3*), and key enzymes involved in the FA biosynthesis pathway, including *FATTY ACID ELONGASE 1* (*FAE1*), *FATTY ACID DESATURASE 2* (*FAD2*) and *FAD3*, was then compared between *BnTT8* double mutants and the corresponding WT during seed development. Relative to WT, the expression of *FUS3*, *FAD2* and *LEC1* was significantly up‐regulated in the *BnTT8* mutant at 14 DAF and/or 28 DAF seeds (Fig. 7). There were no significant changes in the expression of *LEC2*, *FAD3* and *FAE1* when compared to WT and *BnTT8* mutant (Fig. 7). The expression changes in these genes agreed well with the phenotypic variations of oil content and FA composition between WT and the *BnTT8* mutants. Thus, these findings reveal significant roles for *BnTT8* in controlling FA biosynthesis and accumulation.

## Discussion

### The CRISPR/Cas9‐targeted mutations in *BnTT8* is a promising strategy for yellow‐seeded rapeseed breeding

Yellow seed is a desirable trait for *Brassica* oilseed crop breeding due to its better quality than the black‐seeded variety (Marles and Gruber, 2004; Meng *et al.*, 1998; Tang *et al.*, 1997). Unfortunately, no natural yellow‐seeded germplasm has been found in rapeseed, which is recognized as a major obstacle in yellow‐seeded rapeseed breeding (Liu *et al.*, 2005). Thus, the constant creation and use of novel genetic variants are important to the improvement of this trait. To this end, an effective approach is needed to produce targeted mutations in these well‐conserved *tt* homologs in *B. napus*. The newly developed CRISPR/Cas9 technology provides a powerful approach to create novel allelic variation. Thus far, it has been successfully utilized to modify several important agronomic traits in rapeseed, such as multilocular silique, plant height and architecture, and pod shatter resistance, by generating specific gene knockouts (Yang *et al.*, 2017; Yang *et al.*, 2018; Zhai *et al.*, 2019; Zheng *et al.*, 2019). However, no example of the yellow‐seeded improvement in rapeseed via genome editing has been published yet.

In this study, we show the successful utilization of CRISPR/Cas9 for targeted mutations of the *BnTT8* gene in rapeseed with high efficiency. A visible yellow‐seeded knockout phenotype can only be recovered after targeted mutations in both functional copies of the *BnTT8* gene in rapeseed (Table 1; Fig. 3a), which further supports the idea that the role of the *TT8* gene is essential for seed coat colour and is highly conserved in *Brassica* species. Chemical staining of seed coats further confirmed that the specific PA deposition in the seed coat was blocked in the double mutants but not in either of the single mutants (Fig. 3c and d), indicating that the two gene copies have redundant functions in seed colour formation. However, differences between the two *BnTT8* copies were observed, with only the *BnA09.TT8* single mutant resulting in significantly reduced seed coat thickness, whereas the *BnC09.TT8b* single mutant had no significant effects (Fig. 4b). Thus, the two functional copies of the *BnTT8* gene have partially redundant functions in seed coat development, with *BnA09.TT8* making a greater contribution than *BnC09.TT8b*. These observations are in accord with the higher expression level of *BnA09.TT8* than *BnC09.TT8b* during seed development.

The visible yellow‐seeded knockout phenotype was first present in five independent T_0_ transgenic plants and presented the fastest possible scenario in targeted mutations to a polyploid crop. The targeted mutations were stably transmitted to consecutive generations, and a collection of homozygous mutants with loss‐of‐function alleles of the target genes was acquired for phenotyping (Fig. 2c; Table 1). Consistent with previous studies, the seed oil contents of the double mutants steadily increased by 9.47%, 5.89% and 5.80% relative to the corresponding WT seeds from generations T_0_ to T_3_, respectively (Tables 2, 3). Among the selected double mutants, TT8‐96‐3‐2 showed relatively lower oil content (Table 3). Comparison of the mutated locations in these double mutants revealed that TT8‐96‐3‐2 is the only one carrying mutations at the last target (S4) in the *BnTT8* gene (Figs 2c; S5), which may represent a weaker allele mutant and contribute to its weaker phenotypic effect. Since a 1% increase in the seed oil content of rapeseed is equivalent to a 2.3%–2.5% increase in seed yield (Wang, 2004), the yellow‐seeded mutants produced in the present study might offer excellent starting germplasms for promoting high oil production breeding in rapeseed. The protein contents of the double‐mutant seeds were also consistently enhanced across generations (Tables 2 and 3), which were statistically significant in T_0_, T_2_ and two of five T_3_ generations (Tables 2 and 3). The simultaneous increases in both oil and protein contents in the *BnTT8* double mutant were consistent with the advantages of yellow‐seeded *B. napus* over its black‐seeded type reported previously (Marles and Gruber, 2004; Meng *et al.*, 1998; Tang *et al.*, 1997). In contrast, the *tt8* mutation in *Arabidopsis* markedly increased oil content and lowered the protein accumulation of mature seeds (Chen *et al.*, 2014). Thus, *BnTT8* has functionally diverged from its orthologues in *Arabidopsis*. The double mutants also produced seeds with consistently altered FA composition across generations (Tables 2, 3). Assessing the yield‐related traits revealed that these mutants did not cause any severe defects (Table S2), which was consistent with previous reports that most yellow‐seeded materials appear normal morphologically (Yu 2013).

We assayed all potential off‐target loci, and none of them displayed evidence of a CRISPR/Cas9 system‐induced mutation, indicating that the off‐target effect is negligible for well‐designed specific sgRNAs (Table S3). In addition, a variety of transgene‐free *B. napus* plants with homozygous mutations in the target gene were obtained through genetic segregation (Table 1), which would provide valuable resources for rapeseed breeding programmes.

### *BnTT8* is critical for the specific accumulation of PAs in the inner seed coat

In *Brassicaceae*, PAs are the prominent pigments that accumulate especially in the inner integument of the seed coat, where they confer on seeds a dark colour by their oxidation during maturation (Lepiniec *et al.*, 2006; Marles and Gruber, 2004). Based on our vanillin and DMACA assays, it was clearly shown that PA accumulation intensely started at 21 DAF in the black‐seeded WT but not in the yellow‐seeded mutants of *BnTT8* (Fig. 3c and d). Recently, Hong *et al. *(2017) also observed the same phenomenon by chemical staining of developmental seeds in rapeseed, indicating the 21 DAF is an essential stage for seed coat coloration in rapeseed. Consistent with this finding, the expression level of *BnTT8* showed a gradual increase during the early seed developmental stage and peaked at 23 DAF or 35 DAF (Fig. 1), which suggested a positive connection between the expression levels of the *BnTT8* gene and seed coat coloration in rapeseed. Further histological analysis showed that PA accumulation was deposited specifically in the inner integument of the black seed coat (Fig. 4a). However, the disruption of *BnTT8* completely blocked the PA deposition in the seed coat (Fig. 4a), suggesting a critical role for *BnTT8* in the control of the specific accumulation of PAs in the seed coat.

Accumulating evidence indicates that PA biosynthesis in seeds is mainly controlled by the TT8‐involved MBW ternary protein complexes (Xu *et al.*, 2014). In *Arabidopsis*, *TT8* is necessary for normal expression of LBGs by directly binding to their regulatory region but does not affect the expression of EBGs (Xu *et al.*, 2014). Similar observations of the involvement of the *TT8* gene in regulating the expression of LBGs have been reported in *B. rapa* and *B. juncea* (Li *et al.*, 2012a,2012b; Padmaja *et al.*, 2014), which indicated that *TT8* is a regulator of ‘late’ flavonoid metabolism (Nesi *et al.*, 2000). In the present study, transcriptomic profiling revealed that the disruption of *BnTT8* genes reduced not only the expression of LBGs but also that of EBGs, as well as *4CL*, *C4H* and *PAL*, which participated in phenylalanine metabolism (Fig. 5; Table S8). Considering that only *DFR*, *LODX*, *BAN*, *TT19* and *AHA10* in the flavonoid biosynthesis pathways contain cis‐regulatory motifs that can be directly targeted by TT8‐involved complexes (Xu *et al.*, 2014), the broad suppression of phenylpropanoid/flavonoid biosynthesis genes may be the outcome of unknown regulatory mechanisms in rapeseed. Recently, two transcriptomic studies also found that these down‐regulated DEGs in yellow‐seeded coats were enriched in phenylpropanoid and flavonoid biosynthesis in resynthesized yellow‐seeded rapeseed as research materials (Hong *et al.*, 2017; Jiang *et al.*, 2019). Although the causal gene underlying the yellow‐seeded trait is still not clear in their materials, the two functional copies of *BnTT8* were significantly down‐regulated in yellow seed compared with black seed in both cases (Hong *et al.*, 2017; Jiang *et al.*, 2019). It is reasonable to postulate that *BnTT8* is involved in the down‐regulation of these DEGs in phenylpropanoid and flavonoid biosynthesis pathways directly or indirectly. Together, these findings indicate that *BnTT8* plays a key role in the regulation of PA accumulation in the seed coat, and the molecular mechanism underlying seed colour formation in rapeseed is much more complex than that in *Arabidopsis* and other *Brassica* species.

### The mechanism by which *BnTT8* alters oil content and FA composition in seeds

In *Brassica* oil crops, the increased oil content of yellow seeds is widely considered to be a consequence of thinner seed coats because most of the total oil of a seed is synthesized and stored in the embryo (Yu, 2013). Indeed, we observed a thinner seed coat in the double mutant of *BnTT8* relative to WT, which should contribute to its increased oil content (Fig. 4b). Apart from the alteration in the seed coat thickness, the expression of critical TFs involved in seed oil accumulation, including *FUS3* and *LEC1*, was also significantly up‐regulated during seed development in the double mutant of *BnTT8* relative to WT (Fig. 7). *FUS3* and *LEC1*, B3 domain‐containing TFs, are positive regulators of FA biosynthesis during seed maturation (Mu *et al.*, 2008; Yamamoto *et al.*, 2010). Thus, it was possible that the up‐regulation of these genes resulted in the increased oil content in the double mutant of *BnTT8* relative to WT. In *Arabidopsis*, *TT8* can directly bind to the regulatory regions of *LEC1*, *LEC2* and *FUS3* and repress the expression of these genes (Chen *et al.*, 2014). It is most likely that *BnTT8* acted to affect these genes similarly. In addition, it was reported that flavonoids in the seed coat can enter the embryo and participate in inhibiting FA biosynthesis by the repressing of *FabG* and *FabI*, two critical reductases in the FA chain elongation pathway (Zhang and Rock, 2004). The lack of flavonoids in the seed coat could be one of the factors affecting oil content in the *BnTT8* double mutant. In total, the increased oil content in the *BnTT8* double mutant should be the outcome of the joint action of several factors.

In this study, we found that the targeted mutation of the *BnTT8* gene resulted in consistent modification of FA composition across generations, including increases in C16:0, C18:2 and C18:3 and decreases in C18:0 and C18:1 relative to WT seeds (Tables 2and 3). In *Arabidopsis*, the FA composition in seeds is mainly determined by several well‐studied key enzymes, including FAD2 and FAD3, which are involved in FA desaturation, and FAE1, which catalyses the chain elongation process of FA (Baud and Lepiniec, 2009). Our expression analysis of these genes showed that only *FAD2* was up‐regulated significantly in the *BnTT8* mutant (Fig. 7). FAD2 is a key enzyme catalysing the first committed process of the biosynthesis of polyunsaturated FAs from C18:1 to C18:2 (Baud and Lepiniec, 2009). Thus, the up‐regulation of *FAD2* agreed well with the alteration of FA composition in the *BnTT8* mutant. In contrast, the *tt8* mutation in *Arabidopsis* causes significant increases in the expression levels of *FAE1*, *FAD2* and *FAD3* (Chen *et al.*, 2014). Accompanying these DEGs, the *tt8* mutant showed different compositional variations in FA of mature seeds, including increases in C18:1 and decreases in C16:0, C18:2 and C18:3 (Chen *et al.*, 2014). Thus, *BnTT8* appears to have a different function than its orthologues in *Arabidopsis*. Overall, our results reveal a possible mechanism by which *BnTT8* controls seed oil content and FA composition, which needs to be further investigated.

## Materials and methods

### Plant materials

In this study, the *B. napus* pure line J9707 was used as the transformation receptor, and the seeds were obtained from the National Engineering Research Center of Rapeseed, Wuhan, China. Flowers on the primary inflorescence were marked at anthesis, and the seeds at various developmental stages were collected for the following experiments of RNA‐seq, microscopy and chemical staining.

### Construction of the CRISPR/Cas9 vector and plant transformation

The binary pYLCRIPSR/Cas9 multiplex genome targeting vector system was utilized for gene editing in this investigation (Ma *et al.*, 2015). The selection of sequence‐specific sgRNAs in the target gene, CRISPR/Cas9 construct assembly and *Agrobacterium tumefaciens*‐mediated hypocotyl transformation in *B. napus* were conducted as previously described (Yang *et al.*, 2018). The oligos employed in constructing the sgRNA vectors are listed in Table S1. The resulting construct is described in detail in Fig. 2b.

### Identification of transgenic plants and potential off‐targets

The transgenic plants were screened by hygromycin selection (25 mg/L). Then, the presence of the T‐DNA in the construct was assessed by PCR using the specific primer pairs BnTT8T2‐F/PB‐R (Table S1).

The targeted mutations were determined in transgenic plants using the Hi‐TOM platform (Liu *et al.*, 2019). Target‐specific and barcoding PCR, that is two rounds of PCR, were performed to amplify the genomic region encompassing the specific targets of independent samples, and the resulting PCR products were mixed in equal amounts and purified for next‐generation sequencing (the Illumina HiSeq platform at the Novogene Bioinformatics Institute, Beijing, China). The resulting sequencing data were then decoded by a corresponding online tool to track the mutations of the target sites (http://www.hi-tom.net/hi-tom/). The target‐specific primer sets are listed in Table S1.

The potential off‐target sites were identified using CRISPR‐P2.0 (http://crispr.hzau.edu.cn/CRISPR2/). The specific primers that surrounded the potential off‐target sites (Table S1) were used to perform PCR amplification from mixed genomic DNA of 30 T_0_ editing plants. PCR amplification, DNA library construction, sequencing on the Illumina HiSeq 3000 system and data analysis were conducted in accordance with the approach previously described by Yang *et al. *(2018).

### RNA extraction and qRT‐PCR

Total RNA was prepared using the EasyPure Plant RNA Kit (TransGen Biotech, Beijing, China), and cDNA was synthesized using the Transcript RT Kit (TransGen Biotech). qPCR was carried out using the TransStart Top Green qPCR SuperMix Kit (TransGen Biotech) on a CFX384 Real‐Time System (Bio‐Rad). Relative quantification was performed using the comparative cycle threshold method, and the relative amount of PCR product that was amplified using the designed primer sets (Table S1) was normalized to the reference genes *BnPP2A‐1*, *BnACT7* and *BnUBC10*.

### RNA‐seq transcriptomic analysis

Seed coat tissues were sampled with three biological replicates at 14 and 35 DAF. At each stage, seed coats were hand‐dissected from seeds gently on dry ice, immediately frozen in liquid nitrogen and stored at −80 °C until total RNA extraction.

RNA extraction, cDNA library construction, sequencing, quality control, and read mapping to the reference genome, identification of DEGs, and GO and KEGG pathway enrichment analysis of DEGs were performed essentially as described by Shahid *et al. *(2019). Fragments per kilobase of transcript per million mapped reads (FPKM) was calculated as a measure of the level of gene expression. Genes with a false discovery rate (FDR) ≤0.05 and an absolute value of log_2_ fold change ≥1 between mutant and wild type (WT) at each stage were defined as DEGs. The raw sequence data were deposited in the NCBI Sequence Read Archive (Accession No. MN399822).

### Metabolite profiling

Flavonoids were extracted from mature seeds (100 mg dry weight) of the double mutants and WT with six biological replicates and were analysed by LC‐ESI‐MS/MS system at the National Key Laboratory for Crop Genetic Improvement (Huazhong Agricultural University, Wuhan, China). The sample extraction and flavonoid metabolic analysis were done essentially as previously described (Chen *et al.*, 2013).

### Light microscopy

Developing seeds at 28 DAF and 42 DAF were harvested for microscopic analysis. Tissue fixation, embedding, sectioning, and Safranin O and Fast Green staining were performed essentially as described by Li *et al. *(2012a). Images were obtained using a Nikon ECLIPSE 80i compound microscope.

### Vanillin and DMACA staining

The vanillin and DMACA assays were both used for the specific detection of PA accumulation in the developing seed coat. The staining of dissected seed coats was done essentially as previously described (Hong *et al.*, 2017).

### Field experiments and phenotyping

The T_0_ and T_1_ transgenic and WT plants were grown in a greenhouse (16/8 h of light/dark at 22 °C) in 2016 and 2017, respectively. The selected homozygous mutant T_3_ lines without T‐DNA were grown in the winter‐type oilseed rape growing season (2018–2019) on the experimental farm of Huazhong Agriculture University, Wuhan, China. The field experiment followed a randomized complete block design with three replications. Each line was planted in one row with 11–12 plants per row, with a distance of 21 cm between plants within each row and 30 cm between rows. The field management was performed in line with standard breeding practice.

Mature seeds were used for the measurement of seed quality traits, including the protein and oil contents, and FA composition. The FA profiles were determined using gas chromatography (GC) system with a Model 6890 GC analyser (Agilent Technologies, Inc., Wilmington, DE) according to the method previously described by Yang *et al. *(2012). The protein and oil contents were examined using a Foss NIRSystems 5000 near‐infrared reflectance spectroscopy (NIRS) using the parameters described by Gan *et al. *(2003), and the content is expressed as a percentage of the total seed dry weight.

Yield‐related traits, including plant height, branch height, branch number, silique length, number of seeds per silique, 1000‐seed weight and yield/plant, were measured as previously described (Cai *et al.*, 2016).

## Conflict of interest

On behalf of all authors, the corresponding author states that there is no conflict of interest.

## Author contributions

Conceived and designed the experiments: FC, ZY; Performed the experiments: ZY, CS, HL, XL, YY, MB, JY, ZC, SUK; Wrote the manuscript: FC, ZY, YK, AO, MHK; Bioinformatics analysis: YK, ZY.

## Supporting information


**Figure S1** Organization of the predicted TT8 protein indicating the localization of the conserved domains in Arabidopsis and different Brassica species.
**Figure S2** Alignment of TT8 homolog sequences identified from B. napus (BnA09.TT8, BnC09.TT8a and BnC09.TT8b), B. rapa (BrTT8), B. oleracea (BoTT8a and BoTT8b), B. juncea (BjuA.TT8 and BjuB.TT8), and A. thaliana (AtTT8). The base differences are highlighted in grey boxes.
**Figure S3** Sequence alignment of two functional BnTT8 gene copies in J9707.
**Figure S4** Phylogenetic tree showing the sequence relationship among TT8 homologs identified from various plant species.
**Figure S5** The predicted amino acid sequences of BnTT8 homozygous mutants in T2 generation.
**Figure S6** Pearson correlation coefficient among counts of transcriptome data.
**Figure S7** Venn diagrams summarizing the number of differentially expressed genes detected in 14 DAF and 35 DAF seed coats of BnTT8 double mutant (aacc) and WT.
**Figure S8** Number of up‐ and down‐DEGs between BnTT8 double mutant (aacc) and WT identified in developing seeds (14 DAF and 35 DAF).
**Figure S9** Results of GO annotation of all up‐ and down‐regulated genes.
**Figure S10** Results of Top 20 GO annotation of all up‐ and down‐regulated genes.
**Figure S11** Results of KEGG pathway of all up‐ and down‐regulated genes.
**Figure S12** Validation of RNA‐seq data by using qRT‐PCR.

**Table S1** Primers used in the present study.
**Table S2** Yield related traits of WT, the single and double homozygous mutant of BnTT8 without T‐DNA in T3 generation.
**Table S3** Detection of potential off‐target effect at each sgRNA target site.
**Table S4** Statistics of RNA‐seq reads and mapped reads.
**Table S5** DEGs in BnTT8 double mutant (aacc) and WT seed coats at 14 DAF and 35 DAF.
**Table S6** Enriched GO terms of DEGs between BnTT8 double mutant (aacc) and WT during seed development using Blast2GO.
**Table S7** KEGG pathways of DEGs between BnTT8 double mutant (aacc) and WT during seed development.
**Table S8** DEGs related to the Phenylpropanoid and Flavonoid metabolic processes.
**Table S9** Flavonoid metabolites identified in the mature seeds of BnTT8 double mutant (aacc) and its WT control.
**Table S10** Flavonoid metabolites with significant difference between BnTT8 double mutant (aacc) and its WT control.

## References

[pbi13281-bib-0001] Albert, N.W. , Davies, K.M. , Lewis, D.H. , Zhang, H. , Montefiori, M. , Brendolise, C. , Boase, M.R. *et al*. (2014) A conserved network of transcriptional activators and repressors regulates anthocyanin pigmentation in eudicots. Plant Cell, 26, 962–980. 24642943 10.1105/tpc.113.122069 PMC4001404

[pbi13281-bib-0002] Baud, S. and Lepiniec, L. (2009) Regulation of de novo fatty acid synthesis in maturing oilseeds of *Arabidopsis* . Plant Physiol. Biochem. 47, 448–455. 19136270 10.1016/j.plaphy.2008.12.006

[pbi13281-bib-0003] Baudry, A. , Caboche, M. and Lepiniec, L. (2006) TT8 controls its own expression in a feedback regulation involving TTG1 and homologous MYB and bHLH factors, allowing a strong and cell‐specific accumulation of flavonoids in *Arabidopsis thaliana* . Plant J. 46, 768–779. 16709193 10.1111/j.1365-313X.2006.02733.x

[pbi13281-bib-0004] Braatz, J. , Harloff, H.J. , Mascher, M. , Stein, N. , Himmelbach, A. and Jung, C. (2017) CRISPR‐Cas9 targeted mutagenesis leads to simultaneous modification of different homoeologous gene copies in polyploid oilseed rape (*Brassica napus*). Plant Physiol. 174, 935–942. 28584067 10.1104/pp.17.00426 PMC5462057

[pbi13281-bib-0005] Cai, G. , Yang, Q. , Chen, H. , Yang, Q. , Zhang, C. , Fan, C. and Zhou, Y. (2016) Genetic dissection of plant architecture and yield‐related traits in *Brassica napus* . Sci. Rep. 6, 21625. 26880301 10.1038/srep21625 PMC4754947

[pbi13281-bib-0006] Chalhoub, B. , Denoeud, F. , Liu, S. , Parkin, I. , Tang, H. , Wang, X. , Chiquet, J. *et al*. (2014) Early allopolyploid evolution in the post‐Neolithic *Brassica napus* oilseed genome. Science, 345, 950–953. 25146293 10.1126/science.1253435

[pbi13281-bib-0007] Chen, W. , Gong, L. , Guo, Z. , Wang, W. , Zhang, H. , Liu, X. , Yu, S. *et al*. (2013) A novel integrated method for large‐scale detection, identification, and quantification of widely targeted metabolites: application in the study of rice metabolomics. Molecular Plant, 6, 1769–1780. 23702596 10.1093/mp/sst080

[pbi13281-bib-0008] Chen, M. , Xuan, L. , Wang, Z. , Zhou, L. , Li, Z. , Du, X. , Ali, E. *et al*. (2014) *TRANSPARENT TESTA8* inhibits seed fatty acid accumulation by targeting several seed development regulators in *Arabidopsis* . Plant Physiol. 165, 905–916. 24722549 10.1104/pp.114.235507 PMC4044850

[pbi13281-bib-0009] Debeaujon, I. , Nesi, N. , Perez, P. , Devic, M. , Grandjean, O. , Caboche, M. and Lepiniec, L. (2003) Proanthocyanidin‐accumulating cells in Arabidopsis testa: regulation of differentiation and role in seed development. Plant Cell, 15, 2514–2531. 14555692 10.1105/tpc.014043 PMC280558

[pbi13281-bib-0010] Escaray, F.J. , Passeri, V. , Perea‐García, A. , Antonelli, C.J. , Damiani, F. , Ruiz, O.A. and Paolocci, F. (2017) The R2R3‐MYB *TT2b* and the bHLH *TT8* genes are the major regulators of proanthocyanidin biosynthesis in the leaves of *Lotus* species. Planta, 246, 243–261. 28429079 10.1007/s00425-017-2696-6

[pbi13281-bib-0011] Gan, L. , Sun, X. , Jin, L. , Wang, G. , Xu, J. , Wei, Z. and Fu, T. (2003) Establishment of math models of NIRS analysis for oil and protein contents in seed of *Brassica napus* . Sci. Agric. Sin. 36, 1609–1613.

[pbi13281-bib-0012] Hong, M. , Hu, K. , Tian, T. , Li, X. , Chen, L. , Zhang, Y. , Yi, B. *et al*. (2017) Transcriptomic analysis of seed coats in yellow‐seeded *Brassica napus* reveals novel genes that influence proanthocyanidin biosynthesis. Front. Plant Sci. 8, 1674. 29051765 10.3389/fpls.2017.01674 PMC5633857

[pbi13281-bib-0013] Hu, Q. , Hua, W. , Yin, Y. , Zhang, X. , Liu, L. , Shi, J. and Wang, H. (2017) Rapeseed research and production in China. Crop J. 5, 127–135.

[pbi13281-bib-0014] Hu, L. , Zhang, H. , Yang, Q. , Meng, Q. , Han, S. , Nwafor, C.C. , Khan, M.H.U. *et al*. (2018) Promoter variations in a homeobox gene, *BnA10.LMI1*, determine lobed leaves in rapeseed (*Brassica napus* L*.*). Theoret. Appl. Genet. 131, 2699–2708. 30219987 10.1007/s00122-018-3184-5

[pbi13281-bib-0015] Jiang, J. , Zhu, S. , Yuan, Y. , Wang, Y. , Zeng, L. , Batley, J. and Wang, Y.P. (2019) Transcriptomic comparison between developing seeds of yellow‐and black‐seeded *Brassica napus* reveals that genes influence seed quality. BMC Plant Biol. 19, 203. 31096923 10.1186/s12870-019-1821-z PMC6524335

[pbi13281-bib-0016] Lei, Y., Lu, L. , Liu, H.Y. , Li, S. , Xing, F. and Chen, L.L. (2014) CRISPR‐P: a web tool for synthetic single‐guide RNA design of CRISPR‐system in plants. Mol. Plant. 7, 1494–1496. 24719468 10.1093/mp/ssu044

[pbi13281-bib-0017] Lepiniec, L. , Debeaujon, I. , Routaboul, J.M. , Baudry, A. , Pourcel, L. , Nesi, N. and Caboche, M. (2006) Genetics and biochemistry of seed flavonoids. Annu. Rev. Plant Biol. 57, 405–430. 16669768 10.1146/annurev.arplant.57.032905.105252

[pbi13281-bib-0018] Li, A. , Wei, C. , Jiang, J. , Zhang, Y. , Snowdon, R.J. and Wang, Y. (2009) Phenotypic variation in progenies from somatic hybrids between *Brassica napus* and *Sinapis alba* . Euphytica, 170, 289–296.

[pbi13281-bib-0019] Li, X. , Chen, L. , Hong, M. , Zhang, Y. , Zu, F. , Wen, J. , Yi, B. *et al*. (2012a) A large insertion in bHLH transcription factor *BrTT8* resulting in yellow seed coat in *Brassica rapa* . PLoS ONE, 7, 44145. 10.1371/journal.pone.0044145 PMC3439492 22984469

[pbi13281-bib-0020] Li, A. , Jiang, J. , Zhang, Y. , Snowdon, R.J. , Liang, G. and Wang, Y. (2012b) Molecular and cytological characterization of introgression lines in yellow seed derived from somatic hybrids between *Brassica napus* and *Sinapis alba* . Mol. Breed. 29, 209–219.

[pbi13281-bib-0021] Li, P. , Chen, B. , Zhang, G. , Chen, L. , Dong, Q. , Wen, J. , Mysore, K.S. *et al*. (2016) Regulation of anthocyanin and proanthocyanidin biosynthesis by *Medicago truncatula* bHLH transcription factor *MtTT8* . New Phytol. 210, 905–921. 26725247 10.1111/nph.13816

[pbi13281-bib-0022] Li, C. , Hao, M. , Wang, W. , Wang, H. , Chen, F. , Chu, W. and Hu, Q. (2018) An efficient CRISPR/Cas9 platform for rapidly generating simultaneous mutagenesis of multiple gene homoeologs in allotetraploid oilseed rape. Front. Plant Sci. 9, 442. 29731757 10.3389/fpls.2018.00442 PMC5920024

[pbi13281-bib-0023] Li, C. , Qiu, J. , Huang, S. , Yin, J. and Yang, G. (2019) AaMYB3 interacts with AabHLH1 to regulate proanthocyanidin accumulation in *Anthurium andraeanum* (Hort.) – another strategy to modulate pigmentation. Hortic. Res. 6, 14. 30603098 10.1038/s41438-018-0102-6 PMC6312548

[pbi13281-bib-0024] Lian, J. , Lu, X. , Yin, N. , Ma, L. , Lu, J. , Liu, X. , Li, J. *et al*. (2017) Silencing of *BnTT1* family genes affects seed flavonoid biosynthesis and alters seed fatty acid composition in *Brassica napus* . Plant Sci. 254, 32–47. 27964783 10.1016/j.plantsci.2016.10.012

[pbi13281-bib-0025] Lim, S.H. , Kim, D.H. , Kim, J.K. , Lee, J.Y. and Ha, S.H. (2017) A radish basic helix‐loop‐helix transcription factor, *RsTT8* acts a positive regulator for anthocyanin biosynthesis. Front. Plant Sci. 8, 1917. 29167678 10.3389/fpls.2017.01917 PMC5682339

[pbi13281-bib-0026] Liu, X.P. , Tu, J.X. , Chen, B.Y. and Fu, T.D. (2005) Identification and inheritance of a partially dominant gene for yellow seed colour in *Brassica napus* . Plant Breed. 124, 9–12.

[pbi13281-bib-0027] Liu, Q. , Wang, C. , Jiao, X. , Zhang, H. , Song, L. , Li, Y. , Gao, C. *et al*. (2019) Hi‐TOM: a platform for high‐throughput tracking of mutations induced by CRISPR/Cas systems. Sci. China Life Sci. 62, 1–7. 30446870 10.1007/s11427-018-9402-9

[pbi13281-bib-0028] Ma, X. , Zhang, Q. , Zhu, Q. , Liu, W. , Chen, Y. , Qiu, R. , Wang, B. *et al*. (2015) A robust CRISPR/Cas9 system for convenient, high‐efficiency multiplex genome editing in monocot and dicot plants. Mol. Plant, 8, 1274–1284. 25917172 10.1016/j.molp.2015.04.007

[pbi13281-bib-0029] Marles, M.S. and Gruber, M.Y. (2004) Histochemical characterisation of unextractable seed coat pigments and quantification of extractable lignin in the *Brassicaceae* . J. Sci. Food Agric. 84, 251–262.

[pbi13281-bib-0030] Meng, J.L. , Shi, S.W. , Gan, L. , Li, Z.Y. and Qu, X.S. (1998) The production of yellow‐seeded *Brassica napus* (AACC) through crossing interspecific hybrids of *B. campestris* (AA) and *B. carinata* (BBCC) with *B. napus* . Euphytica, 103, 329–333.

[pbi13281-bib-0031] Mu, J. , Tan, H. , Zheng, Q. , Fu, F. , Liang, Y. , Zhang, J. , Yang, X. *et al*. (2008) *LEAFY COTYLEDON1* is a key regulator of fatty acid biosynthesis in *Arabidopsis* . Plant Physiol. 148, 1042–1054. 18689444 10.1104/pp.108.126342 PMC2556827

[pbi13281-bib-0032] Nemesio‐Gorriz, M. , Blair, P.B. , Dalman, K. , Hammerbacher, A. , Arnerup, J. , Stenlid, J. , Mukhtar, S.M. *et al*. (2017) Identification of *Norway spruce* MYB‐bHLH‐WDR transcription factor complex members linked to regulation of the flavonoid pathway. Front. Plant Sci. 8, 305. 28337212 10.3389/fpls.2017.00305 PMC5343035

[pbi13281-bib-0033] Nesi, N. , Debeaujon, I. , Jond, C. , Pelletier, G. , Caboche, M. and Lepiniec, L. (2000) The *TT8* gene encodes a basic helix‐loop‐helix domain protein required for expression of *DFR* and *BAN* genes in *Arabidopsis* siliques. Plant Cell, 12, 1863–1878. 11041882 10.1105/tpc.12.10.1863 PMC149125

[pbi13281-bib-0034] Padmaja, L.K. , Agarwal, P. , Gupta, V. , Mukhopadhyay, A. , Sodhi, Y.S. , Pental, D. and Pradhan, A.K. (2014) Natural mutations in two homoeologous *TT8* genes control yellow seed coat trait in allotetraploid *Brassica juncea* (AABB). Theoret. Appl. Genet. 127, 339–347. 24247234 10.1007/s00122-013-2222-6

[pbi13281-bib-0035] Pourcel, L. , Routaboul, J.M. , Kerhoas, L. , Caboche, M. , Lepiniec, L. and Debeaujon, I. (2005) *TRANSPARENT TESTA10* encodes a laccase‐like enzyme involved in oxidative polymerization of flavonoids in Arabidopsis seed coat. Plant Cell, 17, 2966–2980. 16243908 10.1105/tpc.105.035154 PMC1276023

[pbi13281-bib-0036] Qu, C. , Fu, F. , Lu, K. , Zhang, K. , Wang, R. , Xu, X. , Wang, M. *et al*. (2013) Differential accumulation of phenolic compounds and expression of related genes in black‐and yellow‐seeded *Brassica napus* . J. Exp. Bot. 64, 885–2898. 23698630 10.1093/jxb/ert148 PMC3697950

[pbi13281-bib-0037] Qu, C. , Zhao, H. , Fu, F. , Wang, Z. , Zhang, K. , Zhou, Y. , Wang, X. *et al*. (2016) Genome‐wide survey of flavonoid biosynthesis genes and gene expression analysis between black‐and yellow‐seeded *Brassica napus* . Front. Plant Sci. 7, 1755. 27999578 10.3389/fpls.2016.01755 PMC5139615

[pbi13281-bib-0038] Rahman, M.H. , Joersbo, M. and Poulsen, M.H. (2001) Development of yellow‐seeded *Brassica napus* of double low quality. Plant Breed. 120, 473–478.

[pbi13281-bib-0039] Rashid, A. , Rakow, G. and Downey, R.K. (1994) Development of yellow seeded *Brassica napus* through interspecific crosses. Plant Breed. 112, 127–134.

[pbi13281-bib-0040] Schaart, J.G. , Dubos, C. , Romero De La Fuente, I. , Van Houwelingen, A.M. , de Vos, R.C. , Jonker, H.H. , Xu, W. *et al*. (2013) Identification and characterization of MYB‐bHLH‐WD40 regulatory complexes controlling proanthocyanidin biosynthesis in strawberry (*Fragaria* × *ananassa*) fruits. New Phytol. 197, 454–467. 23157553 10.1111/nph.12017

[pbi13281-bib-0041] Shahid, M. , Cai, G. , Zu, F. , Zhao, Q. , Qasim, M.U. , Hong, Y. , Fan, C. *et al*. (2019) Comparative transcriptome analysis of developing seeds and silique wall reveals dynamic transcription networks for effective oil production in *Brassica napus *L. Int. J. Mol. Sci. 20, 1982. 31018533 10.3390/ijms20081982 PMC6515390

[pbi13281-bib-0042] Shirley, B.W. , Kubasek, W.L. , Storz, G. , Bruggemann, E. , Koornneef, M. , Ausubel, F.M. and Goodman, H.M. (1995) Analysis of Arabidopsis mutants deficient in flavonoid biosynthesis. Plant J. 8, 659–671. 8528278 10.1046/j.1365-313x.1995.08050659.x

[pbi13281-bib-0043] Simbaya, J. , Slominski, B.A. , Rakow, G. , Campbell, L.D. , Downey, R.K. and Bell, J.M. (1995) Quality characteristics of yellow‐seeded *Brassica* seed meals: Protein, carbohydrate, and dietary fiber components. J. Agric. Food Chem. 43, 2062–2066.

[pbi13281-bib-0044] Tang, Z.L. , Li, J.N. , Zhang, X.K. , Chen, L. and Wang, R. (1997) Genetic variation of yellow‐seeded rapeseed lines (*Brassica napus *L.) from different genetic sources. Plant Breed. 116, 471–474.

[pbi13281-bib-0045] Wang, H.Z. (2004) Strategy for rapeseed genetic improvement in China in the coming fifteen years. Chinese J. Oil Crop Sci. 26, 98–101.

[pbi13281-bib-0046] Wang, Y.P. , Sonntag, K. , Rudloff, E. and Chen, J.M. (2005) Intergeneric somatic hybridization between *Brassica napus* L*.* and *Sinapis alba* L. J. Integr. Plant Biol. 47, 84–91.

[pbi13281-bib-0047] Wen, J. , Zhu, L. , Qi, L. , Ke, H. , Yi, B. , Shen, J. , Tu, J. *et al*. (2012) Characterization of interploid hybrids from crosses between *Brassica juncea* and *B. oleracea* and the production of yellow‐seeded *B. napus* . Theoret. Appl. Genet. 125, 19–32. 22350176 10.1007/s00122-012-1813-y

[pbi13281-bib-0048] Woodfield, H.K. , Sturtevant, D. , Borisjuk, L. , Munz, E. , Guschina, I.A. , Chapman, K. and Harwood, J.L. (2017) Spatial and temporal mapping of key lipid species in *Brassica napus* seeds. Plant Physiol. 173, 1998–2009. 28188274 10.1104/pp.16.01705 PMC5373056

[pbi13281-bib-0049] Xu, W. , Grain, D. , Bobet, S. , Le Gourrierec, J. , Thévenin, J. , Kelemen, Z. , Lepiniec, L. *et al*.(2014) Complexity and robustness of the flavonoid transcriptional regulatory network revealed by comprehensive analyses of MYB‐bHLH‐WDR complexes and their targets in *Arabidopsis* seed. New Phytol. 202, 132–144. 24299194 10.1111/nph.12620

[pbi13281-bib-0050] Xu, W. , Dubos, C. and Lepiniec, L. (2015) Transcriptional control of flavonoid biosynthesis by MYB‐bHLH‐WDR complexes. Trends Plant Sci. 20, 176–185. 25577424 10.1016/j.tplants.2014.12.001

[pbi13281-bib-0051] Yamamoto, A. , Kagaya, Y. , Usui, H. , Hobo, T. , Takeda, S. and Hattori, T. (2010) Diverse roles and mechanisms of gene regulation by the Arabidopsis seed maturation master regulator *FUS3* revealed by microarray analysis. Plant Cell Physiol. 51, 2031–2046. 21045071 10.1093/pcp/pcq162

[pbi13281-bib-0052] Yang, Q. , Fan, C. , Guo, Z. , Qin, J. , Wu, J. , Li, Q. , Fu, T. *et al*. (2012) Identification of *FAD2* and *FAD3* genes in *Brassica napus* genome and development of allele‐specific markers for high oleic and low linolenic acid contents. Theoret. Appl. Genet. 125, 715–729. 22534790 10.1007/s00122-012-1863-1

[pbi13281-bib-0053] Yang, H. , Wu, J.J. , Tang, T. , Liu, K.D. and Dai, C. (2017) CRISPR/Cas9‐mediated genome editing efficiently creates specific mutations at multiple loci using one sgRNA in *Brassica napus* . Sci. Rep. 7, 7489. 28790350 10.1038/s41598-017-07871-9 PMC5548805

[pbi13281-bib-0054] Yang, Y. , Zhu, K.Y. , Li, H.L. , Han, S.Q. , Meng, Q.W. , Khan, S.U. , Fan, C. *et al*. (2018) Precise editing of *CLAVATA* genes in *Brassica napus* L. regulates multilocular silique development. Plant Biotechnol. J. 16, 1322–1335. 29250878 10.1111/pbi.12872 PMC5999189

[pbi13281-bib-0055] Yu, C.Y. (2013) Molecular mechanism of manipulating seed coat coloration in oilseed *Brassica* species. J. Appl. Genet. 54, 135–145. 23329015 10.1007/s13353-012-0132-y

[pbi13281-bib-0056] Zhai, Y. , Cai, S. , Hu, L. , Yang, Y. , Amoo, O. , Fan, C. and Zhou, Y. (2019) CRISPR/Cas9‐mediated genome editing reveals differences in the contribution of *INDEHISCENT* homologues to pod shatter resistance in *Brassica napus* L. Theoret. Appl. Genet. 132, 2111–2132. 30980103 10.1007/s00122-019-03341-0

[pbi13281-bib-0057] Zhang, Y.M. and Rock, C.O. (2004) Evaluation of epigallocatechin gallate and related plant polyphenols as inhibitors of the FabG and FabI reductases of bacterial type II fatty‐acid synthase. J. Biol. Chem. 279, 30994–31001. 15133034 10.1074/jbc.M403697200

[pbi13281-bib-0058] Zheng, M. , Zhang, L. , Tang, M. , Liu, J. , Liu, H. , Yang, H. , Fan, S. *et al*. (2019) Knockout of two *BnaMAX1* homologs by CRISPR/Cas9‐targeted mutagenesis improves plant architecture and increases yield in rapeseed (*Brassica napus* L.). Plant Biotechnol. J. 10.1111/pbi.13228. PMC7004912 31373135

