# Supplementary material for: Targeted mutagenesis of BnTT8 homologs controls yellow seed coat development for effective oil production in Brassica napus L
Source: Plant Biotechnol J. 2019 Nov 11;18(5):1153–68. doi: 10.1111/pbi.13281 (PMC7152602; doi:10.1111/pbi.13281)
Supplement: Supplementary file 1 — Figure S1 Organization of the predicted TT8 protein indicating the localization of the conserved domains in Arabidopsis and different Brassica species. Figure S2 Alignment of TT8 homolog sequences identified from B. napus (BnA09.TT8, BnC09.TT8a and BnC09.TT8b), B. rapa (BrTT8), B. oleracea (BoTT8a and BoTT8b), B. juncea (BjuA.TT8 and BjuB.TT8), and A. thaliana (AtTT8). The base differences are highlighted in grey boxes. Figure S3 Sequence alignment of two functional BnTT8 gene copies in J9707. Figure S4 Phylogenetic tree showing the sequence relationship among TT8 homologs identified from various plant species. Figure S5 The predicted amino acid sequences of BnTT8 homozygous mutants in T2 generation. Figure S6 Pearson correlation coefficient among counts of transcriptome data. Figure S7 Venn diagrams summarizing the number of differentially expressed genes detected in 14 DAF and 35 DAF seed coats of BnTT8 double mutant (aacc) and WT. Figure S8 Number of up‐ and down‐DEGs between BnTT8 double mutant (aacc) and WT identified in developing seeds (14 DAF and 35 DAF). Figure S9 Results of GO annotation of all up‐ and down‐regulated genes. Figure S10 Results of Top 20 GO annotation of all up‐ and down‐regulated genes. Figure S11 Results of KEGG pathway of all up‐ and down‐regulated genes. Figure S12 Validation of RNA‐seq data by using qRT‐PCR. [file PBI-18-1153-s002.docx]

**Supplementary Figures**


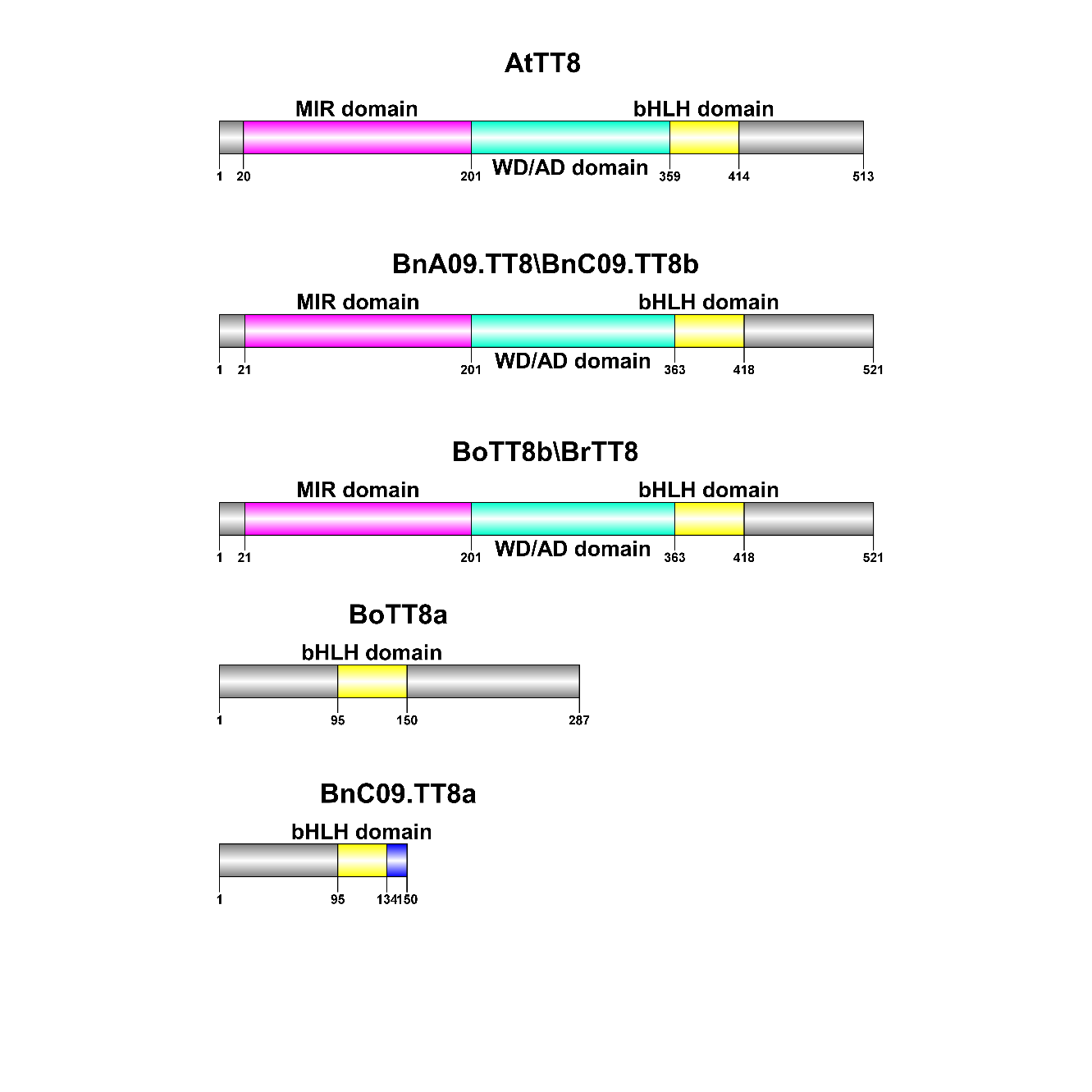


**Fig. S1 Organization of the predicted TT8 protein indicating the localization of the conserved domains in *Arabidopsis* and different *Brassica* species.** MIR, MYB-interacting region; WD/AD, domain of interaction with WD40 and/or with the RNA polymerase II through the acidic domain (AD); bHLH, basic helix-loop-helix domain. Protein sequences are from GenBank with the following accession numbers: AtTT8 (Q9FT81) in *Arabidopsis thaliana*; BoTT8a (VDD31202) and BoTT8b (ADP76654) in *B. oleracea*; BrTT8 ([XP_009113574](https://www.ncbi.nlm.nih.gov/protein/XP_009113574.1?report=genbank&log$=prottop&blast_rank=2&RID=P9CACVHH01R)) in *B. rapa*; BnA09.TT8 (MN399821) and BnC09.TT8b (MN399822) in *B. napus*.


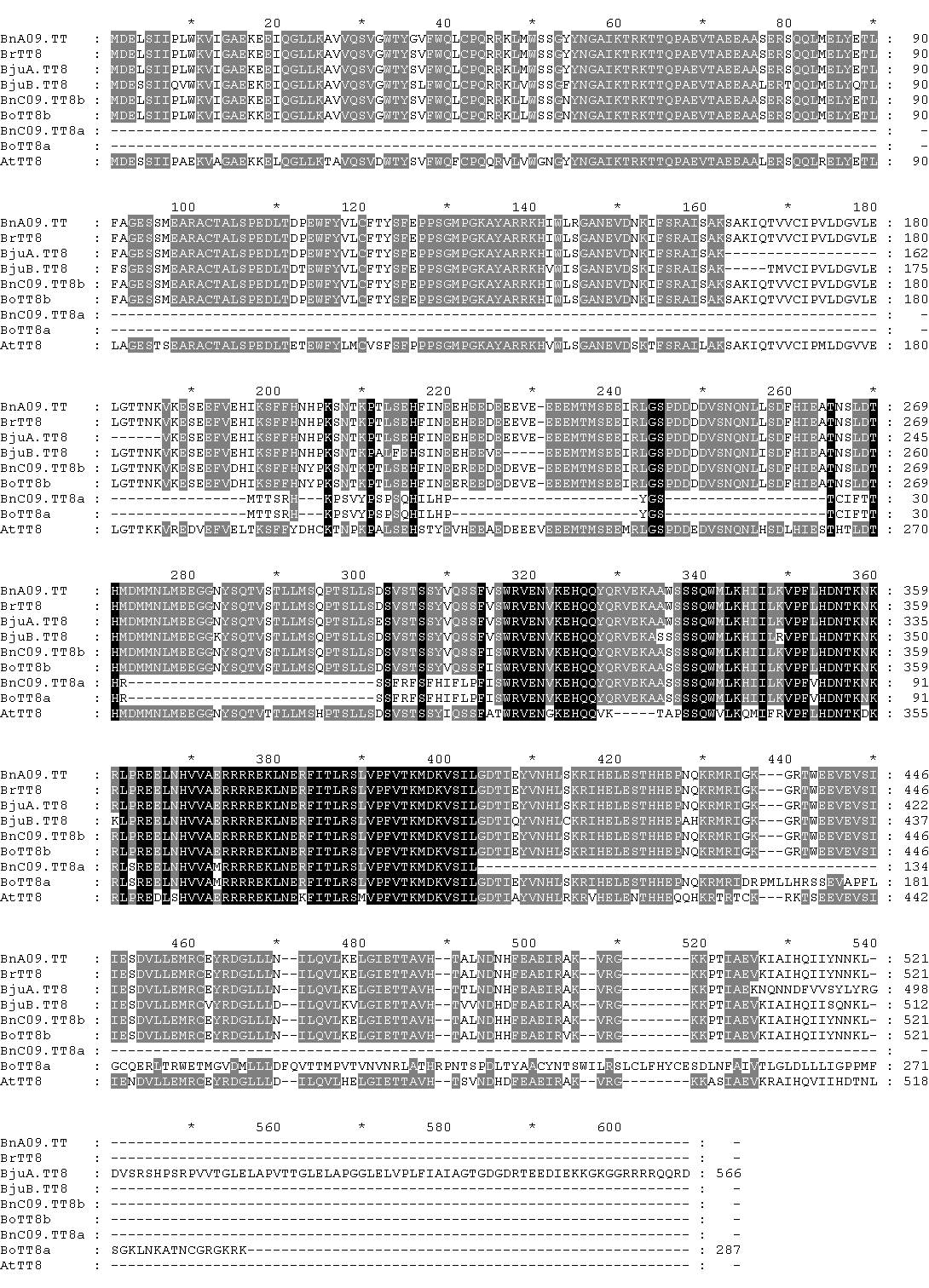


8

8

8

8

8

8

8

MIR domain

bLHL domain

WD/AD domain

**Fig. S2 Alignment of TT8 homolog sequences identified from *B. napus* (BnA09.TT8, BnC09.TT8a and BnC09.TT8b), *B. rapa* (BrTT8), *B. oleracea* (BoTT8a and BoTT8b), *B. juncea* (BjuA.TT8 and BjuB.TT8), and *A. thaliana* (AtTT8). The base differences are highlighted in grey boxes.**

*BnA09.TT8*  ATGGATGAAT TAAGTATTAT ACCGTTATGG AAAGTGATCG GGGCTGAGAA 50

*BnC09.TT8b* ATGGATGAAT TAAGTATTAT ACCGTTATGG AAAGTGATCG GGGCTGAGAA 50

*Clustal*  ********** ********** ********** ********** ********** 50

S1

*BnA09.TT8*  AGAAGAGATT CAAGGGCTAC TTAAGGCGGT GGTGCAATCT GTGGGGTGGA 100

*BnC09.TT8b* AGAAGAGATT CAAGGGCTAC TTAAGGCGGT GGTGCAATCT GTGGGGTGGA 100

*Clustal*  ********** ********** ********** ********** ********** 100

*BnA09.TT8*  CTTATGGTGT CTTCTGGCAA CTTTGTCCTC AACGAAGGTT CACTTTCTTT 150

*BnC09.TT8b* CTTATAGTGT CTTCTGGCAA CTTTGTCCTC AACGAAGGTT C----TCTTT 146

*Clustal*  ***** **** ********** ********** ********** * ***** 145

*BnA09.TT8*  TCATTTCATC CATCTCTCAC AGTATATAAA GCAATATAGT TATCTTTATT 200

*BnC09.TT8b* TCATTTCATC CATCTCTCAC AATATATAAA GCAATATATT TATCTTTATT 196

*Clustal*  ********** ********** * ******** ******** * ********** 193

*BnA09.TT8*  AATTATAATA AGTAGAAGTG ACTAAATGTT AAATCGATTA GGAAATTGAT 250

*BnC09.TT8b* AATTATAATA AGTAGAAGTT ACTAAATGTT AAATCGATTA GGAAATTGTT 246

*Clustal*  ********** ********* ********** ********** ******** * 241

*BnA09.TT8*  GTGGAGTAGT GGATACTACA ACGGCGCAAT AAAGACTAGA AAGACAACTC 300

*BnC09.TT8b* GTGGAGTAGT GGAAACTATA ACGGTGCAAT AAAGACTAGA AAGACAACTC 296

*Clustal*  ********** *** **** * **** ***** ********** ********** 288

S2

*BnA09.TT8*  AGCCGGCGGA AGTTACGGCT GAAGAGGCTG CGTCGGAGAG AAGCCAACAG 350

*BnC09.TT8b* AGCCGGCGGA AGTTACGGCT GAAGAGGCTG CGTCGGAAAG AAGCCAACAG 346

*Clustal*  ********** ********** ********** ******* ** ********** 337

S3

*BnA09.TT8*  CTCATGGAGC TTTACGAGAC GCTTTTTGCT GGAGAATCAT CGATGGAAGC 400

*BnC09.TT8b* CTCATGGAGC TTTACGAGAC GCTTTTTGCT GGAGAATCAT CGATGGAAGC 396

*Clustal*  ********** ********** ********** ********** ********** 387

*BnA09.TT8*  GAGGGCTTGC ACAGCACTGT CGCCGGAGGA TTTGACAGAT CCTGAATGGT 450

*BnC09.TT8b* GAGGGCTTGC ACAGCACTGT CGCCGGAGGA TTTGACGGAT CCTGAATGGT 446

*Clustal*  ********** ********** ********** ****** *** ********** 436

*BnA09.TT8*  TTTATGTGCT GTGTTTCACT TACTCTTTCG AACCTCCTTC TGGGTACAAC 500

*BnC09.TT8b* TTTATGTGCT GTGTTTCACT TACTCTTTCG AACCTCCTTC TGGGTACAAC 496

*Clustal*  ********** ********** ********** ********** ********** 486

*BnA09.TT8*  AACTCTCTCT CTGTCTTCAA AGTTTTTTTT TTCTTTTCAA AAAGACT--A 548

*BnC09.TT8b* AGCTCTCTCT CTGTCTTCAA AGTTTTTTTT T-CTTTTCAA AAAGACTCTA 545

*Clustal*  * ******** ********** ********** * ******** ******* * 532

*BnA09.TT8*  CTCAGAGTTT CTTAATTTGT CTTTTTCATC TTCTCTTAGA GAAGACAAAA 598

*BnC09.TT8b* CCCAGAGTTT CTTAATTTGT CCTTTTCATC TTCTCTTAGA GAAGACAAAA 595

*Clustal*  * ******** ********** * ******** ********** ********** 580

*BnA09.TT8*  ATAGTATTGT GTGTGAAATG CGAATCACAA ATACTATGGA AGCATTAAAG 648

*BnC09.TT8b* ATAGTATTGT GTGTTAAATG TGAATCACAA ATACTACGGA AGCATTAAAG 645

*Clustal*  ********** **** ***** ********* ****** *** ********** 627

*BnA09.TT8*  ACAAACTGGG GAGTTTAAGT TACTGAAAGT AGAAATGTAT TGAAGTTTGT 698

*BnC09.TT8b* ACAAACTGGG GACTTTAAGT TACTGAAAGT AGAAATGTAT TGAATTTAGT 695

*Clustal*  ********** ** ******* ********** ********** **** ** ** 674

*BnA09.TT8*  AAAAACGTAC ACTTCATTTT GGTGAACATA ATTGGACCGT TGAGATTCTT 748

*BnC09.TT8b* GAAAACGTAC ACTTCATTTT GGTGAACATA ATTGGACCGT TGAGATTCTT 745

*Clustal*  ********* ********** ********** ********** ********** 723

*BnA09.TT8*  ATTGGTTTGT TTATTGATTA TCTAAAGTAG GAGCATATAT AGATGATAAA 798

*BnC09.TT8b* ATTGGTTTGT TTATTGATTA TCTAAAGTAG GAGCATATAT AGATGATAAA 795

*Clustal*  ********** ********** ********** ********** ********** 773

*BnA09.TT8*  TGCATATAAA AGTGTGTTAG TTATCGGTAT AATTAATGTT TTTTCTCTAT 848

*BnC09.TT8b* TGCATAACAA AGTGTGGTAG TTATCGGTAT AATTAATGTT TTTTCTCTAT 845

*Clustal*  ****** ** ****** *** ********** ********** ********** 820

*BnA09.TT8*  GGAGGAACAA ATCAAAATAT AATGTGGAAG TATTAATTTG TAGGATGCCA 898

*BnC09.TT8b* GGAGGAAAAA ATCAAAATAT AATGCGGAAG TATTAATTTG TAGGATGCCA 895

*Clustal*  ******* ** ********** **** ***** ********** ********** 868

*BnA09.TT8*  GGAAAAGCGT ATGCGAGGAG GAAGCACATA TGGCTACGTG GTGCAAATGA 948

*BnC09.TT8b* GGAAAGGCGT ATGCGAGGAG GAAGCACATA TGGCTAAGTG GTGCAAATGA 945

*Clustal*  ***** **** ********** ********** ****** *** ********** 916

*BnA09.TT8*  GGTTGACAAT AAAATCTTCT CTAGGGCTAT TTCTGCAAAG GTTCACATC- 997

*BnC09.TT8b* GGTTGACAAT AAAATCTTCT CTAGGGCTAT TTCTGCAAAG GTTTATTTCC 995

*Clustal*  ********** ********** ********** ********** *** * ** 962

*BnA09.TT8*  TTTTATTCAT TCACCACTAC ACTGTGCATC TACTTCTACT TATTTAGATA 1047

*BnC09.TT8b* TTTTATTCAT TCACCACTAC ACTGTGCATC TATTTCTACT TATTTAGATA 1045

*Clustal*  ********** ********** ********** ** ******* ********** 1011

*BnA09.TT8*  TATGCAATTT TATACAT--C TCATTCTGCA AACTAATTAA TTTTATCTTC 1095

*BnC09.TT8b* TACGCAATTT TATATATATC TCATTCTTCA AACTAATTAA TTTTATCTTC 1095

*Clustal*  ** ******* **** ** * ******* ** ********** ********** 1056

*BnA09.TT8*  TTCTTTACTT GAATGCTC-- --TTTCCACA TTAGAGTGCC AAAATTCAGG 1141

*BnC09.TT8b* GTCTTTACTT GAATGCTCGC TCTTTCCACA TTAGAGTGCC AAAATTCAGG 1145

*Clustal*  ********* ******** ******** ********** ********** 1101

*BnA09.TT8*  TAAATTTTGC T-TTATTAAT TATTTATTTA TTTTTCGTAG AAATGAAAGG 1190

*BnC09.TT8b* TAAATTTCGC CGTCATTAAT TAATTATTTA ATTTTCGTAG AAACGAA-GG 1194

*Clustal*  ******* ** * ****** ** ******* ********* *** *** ** 1143

*BnA09.TT8*  TATCAATTAA TAAA------ ---------- ---------- -----GTTAT 1209

*BnC09.TT8b* TATCAATTAA TAAAAGTTAT TTTACATGTT GACAAAAAAG AAGAAGTTAT 1244

*Clustal*  ********** **** ***** 1162

*BnA09.TT8*  TTTACATATT TGACAATTGT TTTGTGATGA AAAAAAAAA- CAAA-AAAAA 1257

*BnC09.TT8b* TTTACATATT TGAGAATTTT TTTGTGATGA AAAAAAAAAA CAAATAAAAA 1294

*Clustal*  ********** *** **** * ********** ********* **** ***** 1208

*BnA09.TT8*  ATCAGACAGT GGTTTGCATT CCCGTGCTTG ATGGCGTTTT GGAACTAGGC 1307

*BnC09.TT8b* AACAGACAGT GGTTTGCATT CCCGTGCTTG ATGGCGTTTT GGAACTAGGC 1344

*Clustal*  * ******** ********** ********** ********** ********** 1257

*BnA09.TT8*  ACAACGAACA AGGTAAAAAT CTCTATTTAT GTCGGTACCC AAAATGTAGT 1357

*BnC09.TT8b* ACAACGAACA AGGTAAAAAT CTCTATTTAT GTCGGTACCC AAAATGTAGA 1394

*Clustal*  ********** ********** ********** ********** ********* 1306

*BnA09.TT8*  CGAATATATT CAGCTCATTC TATATGTTAC TTAGATCATC TCCAAAAAGA 1407

*BnC09.TT8b* CGAATATAGT CAGCTCATTC TA--TGTTAC TTAGA----- ---------- 1427

*Clustal*  ******** * ********** ** ****** ***** 1338

*BnA09.TT8*  CTATATATGG TAAAGTTTCC AAAATTCTAT ATTCAAAGCT TCAAAGTGCT 1457

*BnC09.TT8b* ---------- ---------- ---------- ---------- ---------- 1427

*Clustal*  1338

*BnA09.TT8*  TTTCTCCAAA AACAAAATTT TAAATTTAAC TTCAAAATTA TTTGTAATTT 1507

*BnC09.TT8b* ---------- ---------- ---------- ---------- ---------- 1427

*Clustal*  1338

*BnA09.TT8*  ACAGTATGAT CTTTATACTT ATCATAATTA ATATAAATAC ATAAAACTTT 1557

*BnC09.TT8b* ---------- ---------- ---------- ---------- ---------- 1427

*Clustal*  1338

*BnA09.TT8*  TATAAATAAC TAGCACATAT AAAAAATATT ATAATAATAT TAATTAATAA 1607

*BnC09.TT8b* ---------- ---------- ---------- ---------- ---------- 1427

*Clustal*  1338

*BnA09.TT8*  ATTCTTACAC TAAAATATAA AATTATTAAC AAAAATACAT AATTAAATAT 1657

*BnC09.TT8b* ---------- ---------- ---------- ---------- ---------- 1427

*Clustal*  1338

*BnA09.TT8*  TAAAATACAA GCAAAATATC ACATTAGTCA ATAAAATTAT TTCTGCAATG 1707

*BnC09.TT8b* ---------- ---------- ---------- ---------- ---------- 1427

*Clustal*  1338

*BnA09.TT8*  CTCCATCTTC GGTTACACAA AATTTGTTTG GAAAATATTC TAGAGCTTCT 1757

*BnC09.TT8b* ---------- ---------- ---------- ---------- ---------- 1427

*Clustal*  1338

*BnA09.TT8*  GGAGAAAATT TACTAGACTA TTAGTGTTAT TGTAATATTT AAATTTGTGC 1807

*BnC09.TT8b* ---------- ---------- ---------- ---------- ---------- 1427

*Clustal*  1338

*BnA09.TT8*  AATAACTATG TCTTCATGTA TTTTTTAAAA AATGTTTATT TATTGAGTTT 1857

*BnC09.TT8b* ---------- ---------- ---------- ---------- ---------- 1427

*Clustal*  1338

*BnA09.TT8*  TTTTTGTAAT ATCTTGTTGT GTAATTTTAG TTATAAAATA TTATAAATCT 1907

*BnC09.TT8b* ---------- ---------- ---------- ---------- ---------- 1427

*Clustal*  1338

*BnA09.TT8*  TAACTTAAAA TTTTTATTTA ATTTTATGTG TAAATTTTGA ATTTAAAAAG 1957

*BnC09.TT8b* ---------- ---------- ---------- ---------- ---------- 1427

*Clustal*  1338

*BnA09.TT8*  TAATTTTGAA ATATTTATGA AATAAAAATG TTTTAAAGAT TAATAAGATA 2007

*BnC09.TT8b* ---------- --------GA AATAAAGA-- ---------- ---------- 1437

*Clustal*  ** ****** * 1347

*BnA09.TT8*  AATGAGAAAA TATTTAAAAA TTATTAATAT AATGTGTAAT TAATTAAAGA 2057

*BnC09.TT8b* ---------- ---------- ---------- ---------- ---------- 1437

*Clustal*  1347

*BnA09.TT8*  CCAAAATACA AATAAAAAGA AGAAATTCCA AATTTGGAGT TTTGAGTAGT 2107

*BnC09.TT8b* ---------- ---------- ---------- ---------- ---------- 1437

*Clustal*  1347

*BnA09.TT8*  GAACTTCAAA TATGAAGTTT CATTCTTTAA AACTCTAAAT TCTAAGTTTG 2157

*BnC09.TT8b* ---------- ---------- ---------- ---------- ---------- 1437

*Clustal*  1347

*BnA09.TT8*  AAGTTTTGAA GTTATTTTTT TGAGAGAAAA AACTCTATAT TTGAAGTTAT 2207

*BnC09.TT8b* ---------- ---------- ---------- ---------- ---------- 1437

*Clustal*  1347

*BnA09.TT8*  AGAGTTTCAT TTGGAGATAT TCTTAGAGAA ATAAAAAAAA AAATATACAG 2257

*BnC09.TT8b* ---------- ---------- ---------- ------AAAA TAATATACAG 1451

*Clustal*  **** ********* 1360

*BnA09.TT8*  AAACATCAAC CATTCGCAGT CATATGATAA AAAAATATAG TTTT-CGATT 2306

*BnC09.TT8b* AAACATCAAC CATTCGCAGT CATATGATAA AAAA-TATAG TTTTTCGATT 1500

*Clustal*  ********** ********** ********** **** ***** **** ***** 1408

*BnA09.TT8*  ACATTAAATA CACAACCAAA TTATGTAAAA CTATACAACC TAATGAAAAT 2356

*BnC09.TT8b* ACATTATATA CACAACCAAA TGATGTAAAA -TATACAACC TAATGAAAAT 1549

*Clustal*  ****** *** ********** * ******** ********* ********** 1455

*BnA09.TT8*  ATGCATAAGC GGGAGAACCA GGGAGATGAA TGTATGATAT ATTGTGTTTG 2406

*BnC09.TT8b* ATGCATAAGA GGAAGAACCA GGGAGATGGA TGCATGATAT ATTGTGTTTG 1599

*Clustal*  ********* ** ******* ******** * ** ******* ********** 1501

*BnA09.TT8*  TATGTGAAGG TCAAAGAGAG TGAAGAGTTT GTTGAGCACA TAAAGAGTTT 2456

*BnC09.TT8b* TATGTGAAGG TCAAAGAGAG TGAAGAGTTT GTTGACCACA TAAAGAGTTT 1649

*Clustal*  ********** ********** ********** ***** **** ********** 1550

*BnA09.TT8*  CTTCCACAAC CACCCGAAGT CAAACACTAA GCCTACTCTT TCTGAACACT 2506

*BnC09.TT8b* CTTCCACAAC TACCCGAAGT CAAACACTAA GCCTACTCTT TCTGAACACT 1699

*Clustal*  ********** ********* ********** ********** ********** 1599

*BnA09.TT8*  TCATCAACGA AGAGCATGAA GAAGACGAAG AAGAAGTAGA AGAAGAAGAA 2556

*BnC09.TT8b* TCATCAACGA AGAGCGTGAA GAAGACGAAG ACGAAGTAGA AGAAGAAGAA 1749

*Clustal*  ********** ***** **** ********** * ******** ********** 1647

S4

*BnA09.TT8*  ATGACAATGT CAGAAGAGAT AAGACTTGGT TCTCCTGATG ACGATGACGT 2606

*BnC09.TT8b* ATGACAATGT CAGAGGAGAT AAGACTTGGT TCTCCTGATG ACGATGACGT 1799

*Clustal*  ********** **** ***** ********** ********** ********** 1696

*BnA09.TT8*  CTCCAATCAA AATCTACTCT CTGATTTCCA TATAGAAGCA ACCAATAGTT 2656

*BnC09.TT8b* CTCCAATCAA AATCTACTCT CTGATTTCCA TATAGAAGCA ACCAATAGTT 1849

*Clustal*  ********** ********** ********** ********** ********** 1746

*BnA09.TT8*  TAGGTATACC GTACACACCT TTCTTATTAC ATTAAATTAG TTAACAATAT 2706

*BnC09.TT8b* TAGGTATACC GTACACACCT TTCTTATTAC ATTAAATTAG TTAACAATAT 1899

*Clustal*  ********** ********** ********** ********** ********** 1796

*BnA09.TT8*  CATTATAATT AATTTTCTAA TAATAAATTT TTTAAACTGG TTCTCATGTC 2756

*BnC09.TT8b* CATTATAATA AATTTTCTAA TAATAAATTA TTTAAACTGG TTCTCATGTC 1949

*Clustal*  ********* ********** ********* ********** ********** 1844

*BnA09.TT8*  TGGTAATTCT AACATCTATC ATTGTATAAA TAGATACACA CATGGACATG 2806

*BnC09.TT8b* TGGTAATTCT AACATCTATC ATTGTATATA TAGATACACA CATGGACATG 1999

*Clustal*  ********** ********** ******** * ********** ********** 1893

*BnA09.TT8*  ATGAATCTAA TGGAGGAAGG CGGAAATTAT TCTCAGACAG TATCAACACT 2856

*BnC09.TT8b* ATGAATCTAA TGGAGGAAGG CGGAAATTAT TCTCAGACAG TATCAACACT 2049

*Clustal*  ********** ********** ********** ********** ********** 1943

*BnA09.TT8*  TCTCATGTCA CAACCCACAA GTCTTCTTTC AGATTCAGTT TCCACATCTT 2906

*BnC09.TT8b* TCTCATGTCA CAACCCACCA GTCTTCTTTC AGATTCAGTT TCCACATCTT 2099

*Clustal*  ********** ******** * ********** ********** ********** 1992

*BnA09.TT8*  CTTACGTTCA ATCATCGTTT GTCTCGTGGA GAGTTGAGAA TGTCAAAGAG 2956

*BnC09.TT8b* CTTACGTTCA ATCATCGTTT ATATCGTGGA GAGTTGAGAA TGTCAAAGAG 2149

*Clustal*  ********** ********** * ******* ********** ********** 2040

*BnA09.TT8*  CATCAGCAAT ATCAGCGAGT GGAGAAAGCG GCGTGGTCAT CGTCGCAATG 3006

*BnC09.TT8b* CATCAGCAAT ATCAGCGAGT GGAAAAAGCG GCGTCTTCGT CGTCGCAATG 2199

*Clustal*  ********** ********** *** ****** **** ** * ********** 2086

*BnA09.TT8*  GATGCTCAAA CACATAATCT TGAAAGTTCC TTTCCTCCAC GACAACACTA 3056

*BnC09.TT8b* GATGCTCAAA CACATAATCT TGAAAGTTCC TTTCCTCCAC GACAACACTA 2249

*Clustal*  ********** ********** ********** ********** ********** 2136

*BnA09.TT8*  AAAATAAGAG GCTACCGCGA GAAGAGCTTA ACCATGTGGT GGCCGAGCGA 3106

*BnC09.TT8b* AAAATAAGAG GCTGCCGCGA GAAGAGCTTA ACCATGTGGT GGCCGAGCGA 2299

*Clustal*  ********** *** ****** ********** ********** ********** 2185

*BnA09.TT8*  CGCAGAAGAG AGAAGCTAAA TGAGAGATTC ATAACGTTGA GATCATTGGT 3156

*BnC09.TT8b* CGCAGAAGAG AGAAGCTAAA TGAGAGATTC ATAACGTTGA GATCATTGGT 2349

*Clustal*  ********** ********** ********** ********** ********** 2235

*BnA09.TT8*  TCCATTTGTG ACCAAGATGG ATAAAGTCTC GATCCTTGGA GACACCATTG 3206

*BnC09.TT8b* TCCATTTGTG ACCAAGATGG ATAAAGTCTC GATCCTTGGA GACACCATTG 2399

*Clustal*  ********** ********** ********** ********** ********** 2285

*BnA09.TT8*  AATACGTAAA CCATCTTTCT AAGAGGATAC ATGAGCTGGA ATCTACTCAT 3256

*BnC09.TT8b* AGTACGTAAA CCATCTTTCT AAGAGGATCC ATGAGCTGGA ATCTACTCAT 2449

*Clustal*  * ******** ********** ******** * ********** ********** 2333

*BnA09.TT8*  CACGAGCCAA ACCAAAAGCG GATGCGTATC GGTAAGGGAA GAACTTGGGA 3306

*BnC09.TT8b* CACGAGCCAA ACCAAAAGCG GATGCGTATC GGTAAGGGAA GAACTTGGGA 2499

*Clustal*  ********** ********** ********** ********** ********** 2383

*BnA09.TT8*  AGAGGTGGAG GTTTCCATTA TAGAGAGCGA TGTTTTGTTA GAGATGAGAT 3356

*BnC09.TT8b* AGAGGTGGAG GTTTCCATTA TAGAGAGCGA TGTTTTGTTA GAGATGAGAT 2549

*Clustal*  ********** ********** ********** ********** ********** 2433

*BnA09.TT8*  GCGAGTACCG AGATGGTTTA TTGCTCAACA TTCTTCAGGT ACTTAAGGAG 3406

*BnC09.TT8b* GCGAGTACCG AGATGGTTTA TTGCTCAACA TTCTTCAGGT ACTTAAGGAG 2599

*Clustal*  ********** ********** ********** ********** ********** 2483

*BnA09.TT8*  CTGGGTATAG AGACCACTGC GGTTCACACT GCCTTGAACG ACAATCATTT 3456

*BnC09.TT8b* CTAGGTATAG AGACCACTGC GGTTCACACC GCCTTGAACG ACCACCATTT 2649

*Clustal*  ** ******* ********** ********* ********** ** * ***** 2529

*BnA09.TT8*  TGAGGCAGAG ATAAGGGCGA AAGTGAGAGG GAAGAAACCA ACCATTGCTG 3506

*BnC09.TT8b* TGAGGCAGAG ATAAGGGCGA AAGTGAGAGG GAAGAAACCA ACCATTGCTG 2699

*Clustal*  ********** ********** ********** ********** ********** 2579

*BnA09.TT8*  AGGTTAAAAT AGCCATCCAT CAAATCATAT ATAATAATAA ACTCTAG 3553

*BnC09.TT8b* AGGTTAAAAT AGCCATCCAT CAAATCATAT ATAATAATAA ACTCTAG 2746

*Clustal*  ********** ********** ********** ********** ******* 2626

**Fig. S3 Sequence alignment of two functional *BnTT8* gene copies in J9707. The target sequences are shown in underline with the PAM highlighted in red; and the** **black boxes highlight polymorphism.**





**Fig. S4 Phylogenetic tree showing the sequence relationship among *TT8* homologs identified from various plant species.** Protein sequences are from GenBank with the following accession numbers: AtEGL3 (Q9CAD0), AtGL3 (NP_680372), AtMYC1 (Q8W2F1), and AtTT8 (Q9FT81) in *Arabidopsis thaliana*; BnA09.TT8 (MN399821) and BnC09.TT8b (MN399822) in *Brassica napus*; BoTT8a (VDD31202), BoTT8b (ADP76654) in *Brassica oleracea*; BjuA.TT8 (AIN41653.1) and BjuB.TT8 (ASF79354.1) in *Brassica* *juncea*; BrTT8 ([XP_009113574](https://www.ncbi.nlm.nih.gov/protein/XP_009113574.1?report=genbank&log$=prottop&blast_rank=2&RID=P9CACVHH01R)) in *Brassica rapa*; LjTT8 (AB490778) in *Lotus japonicus*; RsTT8 (KY651179) in *Raphanus sativus*; LtTT8 (KY196485) in *Lotus tenuis*; LcTT8 (KY196477) in *Lotus corniculatus*; AcbHLH42 (QAT77714) in *Actinidia chinensis*; MtTT8 (KM892777) in *Medicago truncatula.*

(a) TT8-96-3-2/TT8-96-3-3 aacc T_2_

>aa

BnA09.TT8 MDELSIIPLWKVIGAEKEEIQGLLKAVVQSVGWTYGVFWQLCPQRRKLMWSSGYYNGAIK 60

BnA09.Taa MDELSIIPLWKVIGAEKEEIQGLLKAVVLWGGLMVSSGNFVLNEGN*

BnA09.TT8 TRKTTQPAEVTAEEAASERSQQLMELYETLFAGESSMEARACTALSPEDLTDPEWFYVLC 120

BnA09.TT8 FTYSFEPPSGMPGKAYARRKHIWLRGANEVDNKIFSRAISAKSAKIQTVVCIPVLDGVLE 180

BnA09.TT8 LGTTNKVKESEEFVEHIKSFFHNHPKSNTKPTLSEHFINEEHEEDEEEVEEEEMTMSEEI 240

BnA09.TT8 RLGSPDDDDVSNQNLLSDFHIEATNSLDTHMDMMNLMEEGGNYSQTVSTLLMSQPTSLLS 300

BnA09.TT8 DSVSTSSYVQSSFVSWRVENVKEHQQYQRVEKAAWSSSQWMLKHIILKVPFLHDNTKNKR 360

BnA09.TT8 LPREELNHVVAERRRREKLNERFITLRSLVPFVTKMDKVSILGDTIEYVNHLSKRIHELE 420

BnA09.TT8 STHHEPNQKRMRIGKGRTWEEVEVSIIESDVLLEMRCEYRDGLLLNILQVLKELGIETTA 480

BnA09.TT8 VHTALNDNHFEAEIRAKVRGKKPTIAEVKIAIHQIIYNNKL* 521

>cc

BnC09.TT8b MDELSIIPLWKVIGAEKEEIQGLLKAVVQSVGWTYSVFWQLCPQRRKLLWSSGNYNGAIK 60

BnA09.Tbcc MDELSIIPLWKVIGAEKEEIQGLLKAVVQSVGWTYSVFWQLCPQRRKLLWSSGNYNGAIK 60

BnC09.TT8b TRKTTQPAEVTAEEAASERSQQLMELYETLFAGESSMEARACTALSPEDLTDPEWFYVLC 120

BnA09.Tbcc TRKTTQPAEVTAEEAASERSQQLMELYETLFAGESSMEARACTALSPEDLTDPEWFYVLC 120

BnC09.TT8b FTYSFEPPSGMPGKAYARRKHIWLSGANEVDNKIFSRAISAKSAKIQTVVCIPVLDGVLE 180

BnA09.Tbcc FTYSFEPPSGMPGKAYARRKHIWLSGANEVDNKIFSRAISAKSAKIQTVVCIPVLDGVLE 180

BnC09.TT8b LGTTNKVKESEEFVDHIKSFFHNYPKSNTKPTLSEHFINEEREEDEDEVEEEEMTMSEEI 240

BnA09.Tbcc LGTTNKVKESEEFVDHIKSFFHNYPKSNTKPTLSEHFINEEREEDEDEVEEEEMTMSEEI 240

BnC09.TT8b RLGSPDDDDVSNQNLLSDFHIEATNSLDTHMDMMNLMEEGGNYSQTVSTLLMSQPTSLLS 300

BnA09.Tbcc RLGSPD*

BnC09.TT8b DSVSTSSYVQSSFISWRVENVKEHQQYQRVEKAASSSSQWMLKHIILKVPFLHDNTKNKR 360

BnC09.TT8b LPREELNHVVAERRRREKLNERFITLRSLVPFVTKMDKVSILGDTIEYVNHLSKRIHELE 420

BnC09.TT8b STHHEPNQKRMRIGKGRTWEEVEVSIIESDVLLEMRCEYRDGLLLNILQVLKELGIETTA 480

BnC09.TT8b VHTALNDHHFEAEIRAKVRGKKPTIAEVKIAIHQIIYNNKL* 521

(b) TT8-148-5-7/TT8-145-13-3 aacc T_2_

>aa

BnA09.TT8 MDELSIIPLWKVIGAEKEEIQGLLKAVVQSVGWTYGVFWQLCPQRRKLMWSSGYYNGAIK 60

BnA09.Taa MDELSIIPLWKVIGAEKEEIQGLLKAVVQSVGWTYGVFWQLCPQRRKLMWSSGYYNGAIK 60

BnA09.TT8 TRKTTQPAEVTAEEAASERSQQLMELYETLFAGESSMEARACTALSPEDLTDPEWFYVLC 120

BnA09.Taa TRKTTQPAEVTAEEAVVGEKPTAHGALRDAFCWRIIDGSEGLHSTVAGGFDRS*

BnA09.TT8 FTYSFEPPSGMPGKAYARRKHIWLRGANEVDNKIFSRAISAKSAKIQTVVCIPVLDGVLE 180

BnA09.TT8 LGTTNKVKESEEFVEHIKSFFHNHPKSNTKPTLSEHFINEEHEEDEEEVEEEEMTMSEEI 240

BnA09.TT8 RLGSPDDDDVSNQNLLSDFHIEATNSLDTHMDMMNLMEEGGNYSQTVSTLLMSQPTSLLS 300

BnA09.TT8 DSVSTSSYVQSSFVSWRVENVKEHQQYQRVEKAAWSSSQWMLKHIILKVPFLHDNTKNKR 360

BnA09.TT8 LPREELNHVVAERRRREKLNERFITLRSLVPFVTKMDKVSILGDTIEYVNHLSKRIHELE 420

BnA09.TT8 STHHEPNQKRMRIGKGRTWEEVEVSIIESDVLLEMRCEYRDGLLLNILQVLKELGIETTA 480

BnA09.TT8 VHTALNDNHFEAEIRAKVRGKKPTIAEVKIAIHQIIYNNKL* 521

>cc

BnC09.TT8b MDELSIIPLWKVIGAEKEEIQGLLKAVVQSVGWTYSVFWQLCPQRRKLLWSSGNYNGAIK 60

BnA09.Tbcc MDELSIIPLWKVIGAEKEEIQGLLKAVVQSCGVDL*

BnC09.TT8b TRKTTQPAEVTAEEAASERSQQLMELYETLFAGESSMEARACTALSPEDLTDPEWFYVLC 120

BnC09.TT8b FTYSFEPPSGMPGKAYARRKHIWLSGANEVDNKIFSRAISAKSAKIQTVVCIPVLDGVLE 180

BnC09.TT8b LGTTNKVKESEEFVDHIKSFFHNYPKSNTKPTLSEHFINEEREEDEDEVEEEEMTMSEEI 240

BnC09.TT8b RLGSPDDDDVSNQNLLSDFHIEATNSLDTHMDMMNLMEEGGNYSQTVSTLLMSQPTSLLS 300

BnC09.TT8b DSVSTSSYVQSSFISWRVENVKEHQQYQRVEKAASSSSQWMLKHIILKVPFLHDNTKNKR 360

BnC09.TT8b LPREELNHVVAERRRREKLNERFITLRSLVPFVTKMDKVSILGDTIEYVNHLSKRIHELE 420

BnC09.TT8b STHHEPNQKRMRIGKGRTWEEVEVSIIESDVLLEMRCEYRDGLLLNILQVLKELGIETTA 480

BnC09.TT8b VHTALNDHHFEAEIRAKVRGKKPTIAEVKIAIHQIIYNNKL* 521

(c) TT8-148-9-2 aacc T_2_

>aa

BnA09.TT8 MDELSIIPLWKVIGAEKEEIQGLLKAVVQSVGWTYGVFWQLCPQRRKLMWSSGYYNGAIK 60

BnA09.Taa MDELSIIPLWKVIGAEKEEIQGLLKAVVQRRLYFFIIRFMDLWCLLATLSSTKEIDVE*

BnA09.TT8 TRKTTQPAEVTAEEAASERSQQLMELYETLFAGESSMEARACTALSPEDLTDPEWFYVLC 120

BnA09.TT8 FTYSFEPPSGMPGKAYARRKHIWLRGANEVDNKIFSRAISAKSAKIQTVVCIPVLDGVLE 180

BnA09.TT8 LGTTNKVKESEEFVEHIKSFFHNHPKSNTKPTLSEHFINEEHEEDEEEVEEEEMTMSEEI 240

BnA09.TT8 RLGSPDDDDVSNQNLLSDFHIEATNSLDTHMDMMNLMEEGGNYSQTVSTLLMSQPTSLLS 300

BnA09.TT8 DSVSTSSYVQSSFVSWRVENVKEHQQYQRVEKAAWSSSQWMLKHIILKVPFLHDNTKNKR 360

BnA09.TT8 LPREELNHVVAERRRREKLNERFITLRSLVPFVTKMDKVSILGDTIEYVNHLSKRIHELE 420

BnA09.TT8 STHHEPNQKRMRIGKGRTWEEVEVSIIESDVLLEMRCEYRDGLLLNILQVLKELGIETTA 480

BnA09.TT8 VHTALNDNHFEAEIRAKVRGKKPTIAEVKIAIHQIIYNNKL* 521

>cc

BnC09.TT8b MDELSIIPLWKVIGAEKEEIQGLLKAVVQSVGWTYSVFWQLCPQRRKLLWSSGNYNGAIK 60

BnA09.Tbcc MDELSIIPLWKVIGAEKEEIQGLLKAVVLWGGLIVSSGNFVLNEGNCCGVVETITVQ*

BnC09.TT8b TRKTTQPAEVTAEEAASERSQQLMELYETLFAGESSMEARACTALSPEDLTDPEWFYVLC 120

BnC09.TT8b FTYSFEPPSGMPGKAYARRKHIWLSGANEVDNKIFSRAISAKSAKIQTVVCIPVLDGVLE 180

BnC09.TT8b LGTTNKVKESEEFVDHIKSFFHNYPKSNTKPTLSEHFINEEREEDEDEVEEEEMTMSEEI 240

BnC09.TT8b RLGSPDDDDVSNQNLLSDFHIEATNSLDTHMDMMNLMEEGGNYSQTVSTLLMSQPTSLLS 300

BnC09.TT8b DSVSTSSYVQSSFISWRVENVKEHQQYQRVEKAASSSSQWMLKHIILKVPFLHDNTKNKR 360

BnC09.TT8b LPREELNHVVAERRRREKLNERFITLRSLVPFVTKMDKVSILGDTIEYVNHLSKRIHELE 420

BnC09.TT8b STHHEPNQKRMRIGKGRTWEEVEVSIIESDVLLEMRCEYRDGLLLNILQVLKELGIETTA 480

BnC09.TT8b VHTALNDHHFEAEIRAKVRGKKPTIAEVKIAIHQIIYNNKL* 521

(d) TT8-281-5-5 aacc T_2_

>aa

BnA09.TT8 MDELSIIPLWKVIGAEKEEIQGLLKAVVQSVGWTYGVFWQLCPQRRKLMWSSGYYNGAIK 60

BnA09.Taa MDELSIIPLWKVIGAEKEEIQGLLKAVVQSCGVDLWCLLATLSSTKEIDVE*

BnA09.TT8 TRKTTQPAEVTAEEAASERSQQLMELYETLFAGESSMEARACTALSPEDLTDPEWFYVLC 120

BnA09.TT8 FTYSFEPPSGMPGKAYARRKHIWLRGANEVDNKIFSRAISAKSAKIQTVVCIPVLDGVLE 180

BnA09.TT8 LGTTNKVKESEEFVEHIKSFFHNHPKSNTKPTLSEHFINEEHEEDEEEVEEEEMTMSEEI 240

BnA09.TT8 RLGSPDDDDVSNQNLLSDFHIEATNSLDTHMDMMNLMEEGGNYSQTVSTLLMSQPTSLLS 300

BnA09.TT8 DSVSTSSYVQSSFVSWRVENVKEHQQYQRVEKAAWSSSQWMLKHIILKVPFLHDNTKNKR 360

BnA09.TT8 LPREELNHVVAERRRREKLNERFITLRSLVPFVTKMDKVSILGDTIEYVNHLSKRIHELE 420

BnA09.TT8 STHHEPNQKRMRIGKGRTWEEVEVSIIESDVLLEMRCEYRDGLLLNILQVLKELGIETTA 480

BnA09.TT8 VHTALNDNHFEAEIRAKVRGKKPTIAEVKIAIHQIIYNNKL* 521

>cc

BnC09.TT8b MDELSIIPLWKVIGAEKEEIQGLLKAVVQSVGWTYSVFWQLCPQRRKLLWSSGNYNGAIK 60

BnC09.Tbcc MDELSIIPLWKVIGAEKEEIQGLLKAVVQS--WTYSVFWQLCPQRRKLLWSSGNYNGAIK 58

BnC09.TT8b TRKTTQPAEVTAEEAASERSQQLMELYETLFAGESSMEARACTALSPEDLTDPEWFYVLC 120

BnC09.Tbcc TRKTTQPAEVTAEEAASERSQQLMELYETLFAGESSMEARACTALSPEDLTDPEWFYVLC 118

BnC09.TT8b FTYSFEPPSGMPGKAYARRKHIWLSGANEVDNKIFSRAISAKSAKIQTVVCIPVLDGVLE 180

BnC09.Tbcc FTYSFEPPSGMPGKAYARRKHIWLSGANEVDNKIFSRAISAKSAKIQTVVCIPVLDGVLE 178

BnC09.TT8b LGTTNKVKESEEFVDHIKSFFHNYPKSNTKPTLSEHFINEEREEDEDEVEEEEMTMSEEI 240

BnC09.Tbcc LGTTNKVKESEEFVDHIKSFFHNYPKSNTKPTLSEHFINEEREEDEDEVEEEEMTMSEEI 238

BnC09.TT8b RLGSPDDDDVSNQNLLSDFHIEATNSLDTHMDMMNLMEEGGNYSQTVSTLLMSQPTSLLS 300

BnC09.Tbcc RLGSPDDDDVSNQNLLSDFHIEATNSLDTHMDMMNLMEEGGNYSQTVSTLLMSQPTSLLS 298

BnC09.TT8b DSVSTSSYVQSSFISWRVENVKEHQQYQRVEKAASSSSQWMLKHIILKVPFLHDNTKNKR 360

BnC09.Tbcc DSVSTSSYVQSSFISWRVENVKEHQQYQRVEKAASSSSQWMLKHIILKVPFLHDNTKNKR 358

BnC09.TT8b LPREELNHVVAERRRREKLNERFITLRSLVPFVTKMDKVSILGDTIEYVNHLSKRIHELE 420

BnC09.Tbcc LPREELNHVVAERRRREKLNERFITLRSLVPFVTKMDKVSILGDTIEYVNHLSKRIHELE 418

BnC09.TT8b STHHEPNQKRMRIGKGRTWEEVEVSIIESDVLLEMRCEYRDGLLLNILQVLKELGIETTA 480

BnC09.Tbcc STHHEPNQKRMRIGKGRTWEEVEVSIIESDVLLEMRCEYRDGLLLNILQVLKELGIETTA 478

BnC09.TT8b VHTALNDHHFEAEIRAKVRGKKPTIAEVKIAIHQIIYNNKL* 521

BnC09.Tbcc VHTALNDHHFEAEIRAKVRGKKPTIAEVKIAIHQIIYNNKL* 519

(e) TT8-299-12-2/ TT8-270-1-9/TT8-299-9-10/TT8-299-12-4 aacc T_2_

>aa

BnA09.TT8 MDELSIIPLWKVIGAEKEEIQGLLKAVVQSVGWTYGVFWQLCPQRRKLMWSSGYYNGAIK 60

BnA09.Taa MDELSIIPLWKVIGAEKEEIQGLLKAVVQSPWSFTRRFLLENHRWKRGLAQHCRRRI*

BnA09.TT8 TRKTTQPAEVTAEEAASERSQQLMELYETLFAGESSMEARACTALSPEDLTDPEWFYVLC 120

BnA09.TT8 FTYSFEPPSGMPGKAYARRKHIWLRGANEVDNKIFSRAISAKSAKIQTVVCIPVLDGVLE 180

BnA09.TT8 LGTTNKVKESEEFVEHIKSFFHNHPKSNTKPTLSEHFINEEHEEDEEEVEEEEMTMSEEI 240

BnA09.TT8 RLGSPDDDDVSNQNLLSDFHIEATNSLDTHMDMMNLMEEGGNYSQTVSTLLMSQPTSLLS 300

BnA09.TT8 DSVSTSSYVQSSFVSWRVENVKEHQQYQRVEKAAWSSSQWMLKHIILKVPFLHDNTKNKR 360

BnA09.TT8 LPREELNHVVAERRRREKLNERFITLRSLVPFVTKMDKVSILGDTIEYVNHLSKRIHELE 420

BnA09.TT8 STHHEPNQKRMRIGKGRTWEEVEVSIIESDVLLEMRCEYRDGLLLNILQVLKELGIETTA 480

BnA09.TT8 VHTALNDNHFEAEIRAKVRGKKPTIAEVKIAIHQIIYNNKL* 521

>cc

BnC09.TT8b MDELSIIPLWKVIGAEKEEIQGLLKAVVQSVGWTYSVFWQLCPQRRKLLWSSGNYNGAIK 60

BnA09.Tbcc MDELSIIPLWKVIGAEKEEIQGLLKAVVQSCGVDL*

BnC09.TT8b TRKTTQPAEVTAEEAASERSQQLMELYETLFAGESSMEARACTALSPEDLTDPEWFYVLC 120

BnC09.TT8b FTYSFEPPSGMPGKAYARRKHIWLSGANEVDNKIFSRAISAKSAKIQTVVCIPVLDGVLE 180

BnC09.TT8b LGTTNKVKESEEFVDHIKSFFHNYPKSNTKPTLSEHFINEEREEDEDEVEEEEMTMSEEI 240

BnC09.TT8b RLGSPDDDDVSNQNLLSDFHIEATNSLDTHMDMMNLMEEGGNYSQTVSTLLMSQPTSLLS 300

BnC09.TT8b DSVSTSSYVQSSFISWRVENVKEHQQYQRVEKAASSSSQWMLKHIILKVPFLHDNTKNKR 360

BnC09.TT8b LPREELNHVVAERRRREKLNERFITLRSLVPFVTKMDKVSILGDTIEYVNHLSKRIHELE 420

BnC09.TT8b STHHEPNQKRMRIGKGRTWEEVEVSIIESDVLLEMRCEYRDGLLLNILQVLKELGIETTA 480

BnC09.TT8b VHTALNDHHFEAEIRAKVRGKKPTIAEVKIAIHQIIYNNKL* 521

(f) TT8-384-4-10/TT8-384-13-3 aacc T_2_

>aa

BnA09.TT8 MDELSIIPLWKVIGAEKEEIQGLLKAVVQSVGWTYGVFWQLCPQRRKLMWSSGYYNGAIK 60

BnA09.Taa MDELSIIPLWKVIGAEKEEIQGLLKAVVQSVGWTYGVFWQLCPQRRKLMWSSGYYNGAIK 60

BnA09.TT8 TRKTTQPAEVTAEEAASERSQQLMELYETLFAGESSMEARACTALSPEDLTDPEWFYVLC 120

BnA09.Taa TRKTTQPAEVTAEEAGVGEKPTAHGALRDAFCWRIIDGSEGLHSTVAGGFDRS*

BnA09.TT8 FTYSFEPPSGMPGKAYARRKHIWLRGANEVDNKIFSRAISAKSAKIQTVVCIPVLDGVLE 180

BnA09.TT8 LGTTNKVKESEEFVEHIKSFFHNHPKSNTKPTLSEHFINEEHEEDEEEVEEEEMTMSEEI 240

BnA09.TT8 RLGSPDDDDVSNQNLLSDFHIEATNSLDTHMDMMNLMEEGGNYSQTVSTLLMSQPTSLLS 300

BnA09.TT8 DSVSTSSYVQSSFVSWRVENVKEHQQYQRVEKAAWSSSQWMLKHIILKVPFLHDNTKNKR 360

BnA09.TT8 LPREELNHVVAERRRREKLNERFITLRSLVPFVTKMDKVSILGDTIEYVNHLSKRIHELE 420

BnA09.TT8 STHHEPNQKRMRIGKGRTWEEVEVSIIESDVLLEMRCEYRDGLLLNILQVLKELGIETTA 480

BnA09.TT8 VHTALNDNHFEAEIRAKVRGKKPTIAEVKIAIHQIIYNNKL* 521

BnC09.TT8b MDELSIIPLWKVIGAEKEEIQGLLKAVVQSVGWTYSVFWQLCPQRRKLLWSSGNYNGAIK 60

BnA09.Tbcc MDELSIIPLWKVIGAEKEEIQGLLKAVVLWGGLIVSSGNFVLNEGNCCGVVETITVQ*

BnC09.TT8b TRKTTQPAEVTAEEAASERSQQLMELYETLFAGESSMEARACTALSPEDLTDPEWFYVLC 120

BnC09.TT8b FTYSFEPPSGMPGKAYARRKHIWLSGANEVDNKIFSRAISAKSAKIQTVVCIPVLDGVLE 180

BnC09.TT8b LGTTNKVKESEEFVDHIKSFFHNYPKSNTKPTLSEHFINEEREEDEDEVEEEEMTMSEEI 240

BnC09.TT8b RLGSPDDDDVSNQNLLSDFHIEATNSLDTHMDMMNLMEEGGNYSQTVSTLLMSQPTSLLS 300

BnC09.TT8b DSVSTSSYVQSSFISWRVENVKEHQQYQRVEKAASSSSQWMLKHIILKVPFLHDNTKNKR 360

BnC09.TT8b LPREELNHVVAERRRREKLNERFITLRSLVPFVTKMDKVSILGDTIEYVNHLSKRIHELE 420

BnC09.TT8b STHHEPNQKRMRIGKGRTWEEVEVSIIESDVLLEMRCEYRDGLLLNILQVLKELGIETTA 480

BnC09.TT8b VHTALNDHHFEAEIRAKVRGKKPTIAEVKIAIHQIIYNNKL* 521

(g) TT8-291-6-4/TT8-291-9-7 aaCC T_2_

>aa

BnA09.TT8 MDELSIIPLWKVIGAEKEEIQGLLKAVVQSVGWTYGVFWQLCPQRRKLMWSSGYYNGAIK 60

BnA09.Taa MDELSIIPLWKVIGAEKEEIQGLLKAVVQSVGWTYGVFWQLCPQRRKLMWSSGYYNGAIK 60

BnA09.TT8 TRKTTQPAEVTAEEAASERSQQLMELYETLFAGESSMEARACTALSPEDLTDPEWFYVLC 120

BnA09.Taa TRKTTQPAEVTAEEADVGEKPTAHGALRDAFCWRIIDGSEGLHSTVAGGFDRS*

BnA09.TT8 FTYSFEPPSGMPGKAYARRKHIWLRGANEVDNKIFSRAISAKSAKIQTVVCIPVLDGVLE 180

BnA09.TT8 LGTTNKVKESEEFVEHIKSFFHNHPKSNTKPTLSEHFINEEHEEDEEEVEEEEMTMSEEI 240

BnA09.TT8 RLGSPDDDDVSNQNLLSDFHIEATNSLDTHMDMMNLMEEGGNYSQTVSTLLMSQPTSLLS 300

BnA09.TT8 DSVSTSSYVQSSFVSWRVENVKEHQQYQRVEKAAWSSSQWMLKHIILKVPFLHDNTKNKR 360

BnA09.TT8 LPREELNHVVAERRRREKLNERFITLRSLVPFVTKMDKVSILGDTIEYVNHLSKRIHELE 420

BnA09.TT8 STHHEPNQKRMRIGKGRTWEEVEVSIIESDVLLEMRCEYRDGLLLNILQVLKELGIETTA 480

BnA09.TT8 VHTALNDNHFEAEIRAKVRGKKPTIAEVKIAIHQIIYNNKL* 521

(h) TT8-270-11-3/TT8-270-11-5/TT8-384-14-10 AAcc T_2_

>cc

BnC09.TT8b MDELSIIPLWKVIGAEKEEIQGLLKAVVQSVGWTYSVFWQLCPQRRKLLWSSGNYNGAIK 60

BnA09.Tbcc MDELSIIPLWKVIGAEKEEIQGLLKAVVQSCGVDL*

BnC09.TT8b TRKTTQPAEVTAEEAASERSQQLMELYETLFAGESSMEARACTALSPEDLTDPEWFYVLC 120

BnC09.TT8b FTYSFEPPSGMPGKAYARRKHIWLSGANEVDNKIFSRAISAKSAKIQTVVCIPVLDGVLE 180

BnC09.TT8b LGTTNKVKESEEFVDHIKSFFHNYPKSNTKPTLSEHFINEEREEDEDEVEEEEMTMSEEI 240

BnC09.TT8b RLGSPDDDDVSNQNLLSDFHIEATNSLDTHMDMMNLMEEGGNYSQTVSTLLMSQPTSLLS 300

BnC09.TT8b DSVSTSSYVQSSFISWRVENVKEHQQYQRVEKAASSSSQWMLKHIILKVPFLHDNTKNKR 360

BnC09.TT8b LPREELNHVVAERRRREKLNERFITLRSLVPFVTKMDKVSILGDTIEYVNHLSKRIHELE 420

BnC09.TT8b STHHEPNQKRMRIGKGRTWEEVEVSIIESDVLLEMRCEYRDGLLLNILQVLKELGIETTA 480

BnC09.TT8b VHTALNDHHFEAEIRAKVRGKKPTIAEVKIAIHQIIYNNKL* 521

(i) TT8-384-14-9 AAcc T_2_

>cc

BnC09.TT8b MDELSIIPLWKVIGAEKEEIQGLLKAVVQSVGWTYSVFWQLCPQRRKLLWSSGNYNGAIK 60

BnA09.Tbcc MDELSIIPLWKVIGAEKEEIQGLLKAVVQLCGVDL*

BnC09.TT8b TRKTTQPAEVTAEEAASERSQQLMELYETLFAGESSMEARACTALSPEDLTDPEWFYVLC 120

BnC09.TT8b FTYSFEPPSGMPGKAYARRKHIWLSGANEVDNKIFSRAISAKSAKIQTVVCIPVLDGVLE 180

BnC09.TT8b LGTTNKVKESEEFVDHIKSFFHNYPKSNTKPTLSEHFINEEREEDEDEVEEEEMTMSEEI 240

BnC09.TT8b RLGSPDDDDVSNQNLLSDFHIEATNSLDTHMDMMNLMEEGGNYSQTVSTLLMSQPTSLLS 300

BnC09.TT8b DSVSTSSYVQSSFISWRVENVKEHQQYQRVEKAASSSSQWMLKHIILKVPFLHDNTKNKR 360

BnC09.TT8b LPREELNHVVAERRRREKLNERFITLRSLVPFVTKMDKVSILGDTIEYVNHLSKRIHELE 420

BnC09.TT8b STHHEPNQKRMRIGKGRTWEEVEVSIIESDVLLEMRCEYRDGLLLNILQVLKELGIETTA 480

BnC09.TT8b VHTALNDHHFEAEIRAKVRGKKPTIAEVKIAIHQIIYNNKL* 521

**Fig. S5** The predicted amino acid sequences of *BnTT8* homozygous mutants in T_2_ generation. TT8-96-3-2/TT8-96-3-3 (a), TT8-148-5-7/TT8-145-13-3 (b), TT8-148-9-2 (c), TT8-281-5-5 (d), TT8-299-12-2/TT8-270-1-9/TT8-299-9-10/TT8-299-12-4 (e), TT8-384-4-10/TT8-384-13-3 (f), are double-homozygous mutant lines of *BnTT8*; TT8-291-6-4/TT8-291-9-7 (g) are single-homozygous mutant lines of *BnA09.TT8*; TT8-270-11-3/TT8-270-11-5/TT8-384-14-10 (h), TT8-384-14-9 (i) are single-homozygous mutant lines of *BnC09.TT8b*. Stars indicate stop codon and numbers indicate amino acid positions; red letters indicate frame-shift amino acids; “-” indicate deletion of amino acid; “aa” and “cc” represents the homozygous mutated alleles of the target gene on *BnA09.TT8* and *BnC09.TT8b*, respectively; “aaCC”, “AAcc” and “aacc” represent homozygous mutations of the target gene in *BnA09.TT8*, *BnC09.TT8b* and both copies, respectively.


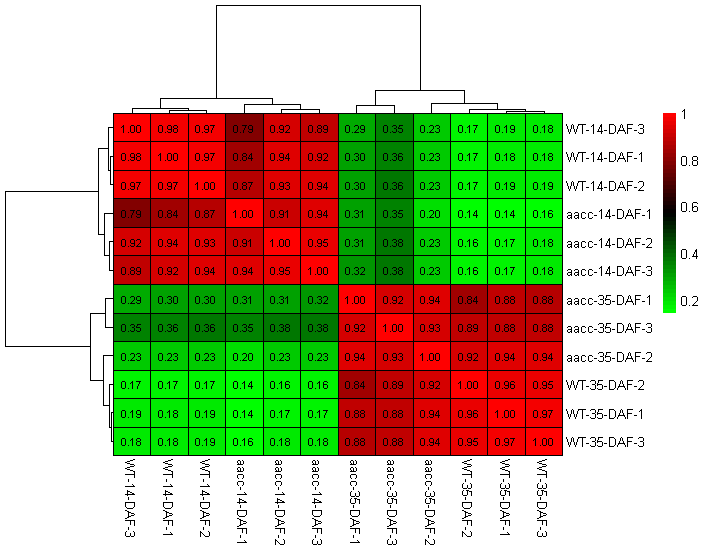


**Fig. S6 Pearson correlation coefficient among counts of transcriptome data.**


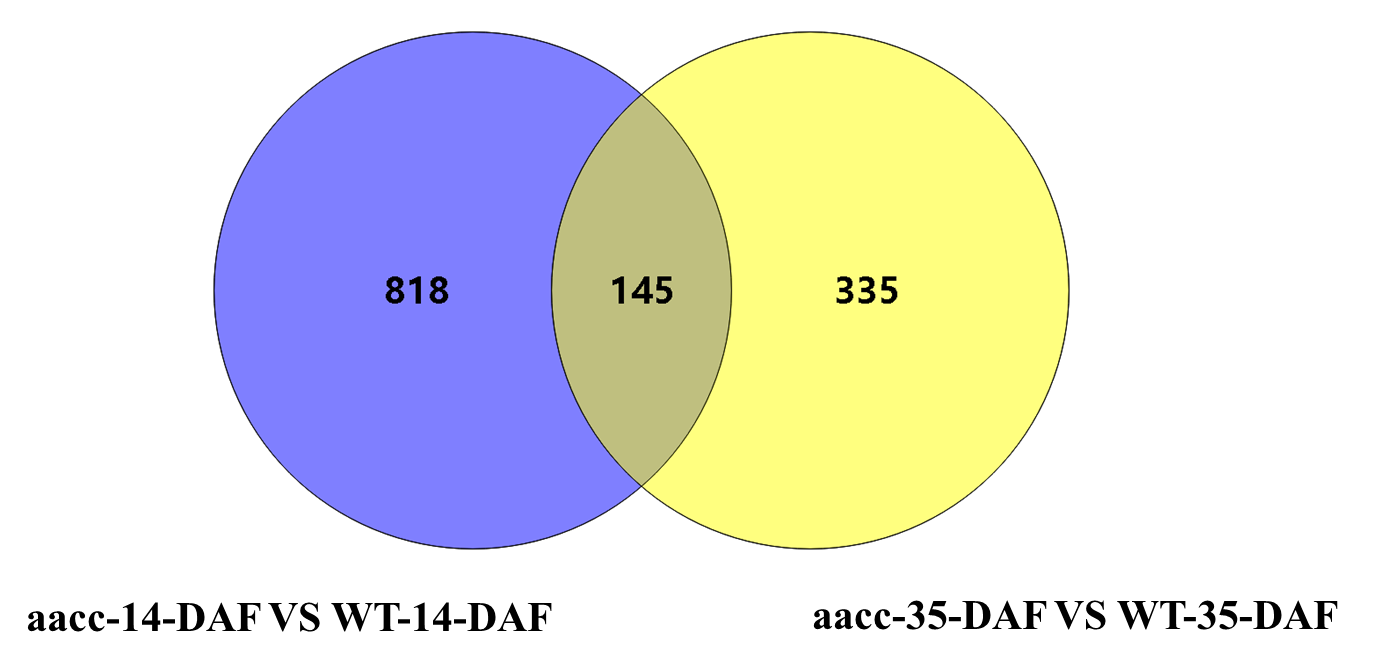


aacc (14 DAF) vs WT (14 DAF) aacc (35 DAF) vs WT (35 DAF)

**Fig. S7 Venn diagrams summarizing the number differentially expressed genes detected in 14 DAF and 35 DAF seed coats of *BnTT8* double mutant (aacc) and WT.**

**Fig. S8 Number of up- and down-DEGs between *BnTT8* double mutant (aacc) and WT identified in developing seeds (14 DAF and 35 DAF).**

(a)


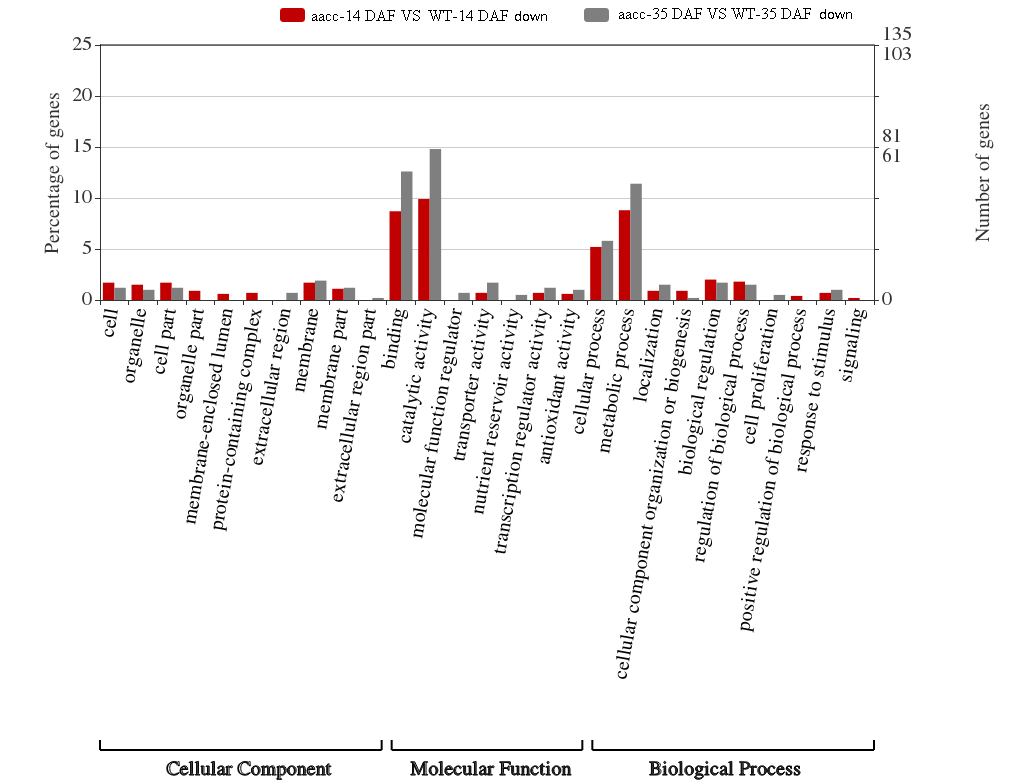


(b)


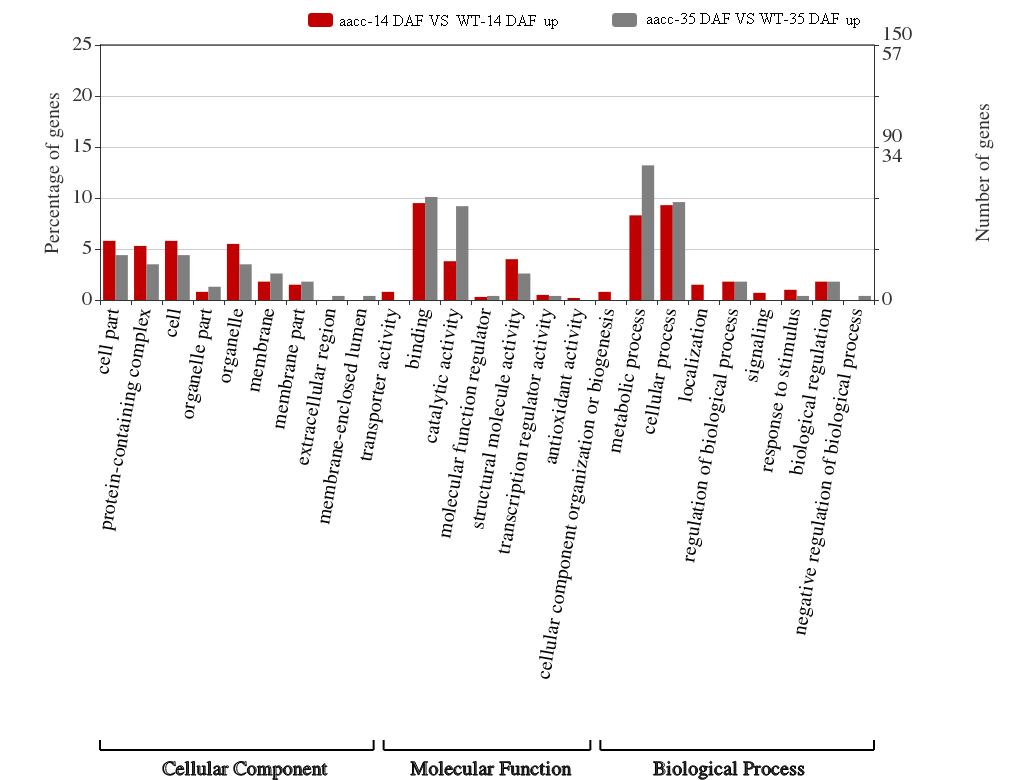


**Fig. S9** **Results of GO annotation of all up- and down-regulated genes.** (a) GO Annotation Map of down-regulated Genes (b) GO Annotation Map of up-regulated Genes.

(a)

(b)

(c)

(d)

**Fig. S10** **Results of Top 20 GO annotation of all up- and down-regulated genes.** (a) Top 20 GO Annotation Map of down-DEGs in 14 DAF; (b) Top 20 GO Annotation Map of down-DEGs in 35 DAF; (c) Top 20 GO Annotation Map of up-DEGs in 14 DAF; (d) Top 20 GO Annotation Map of up-DEGs in 35 DAF.

(a)


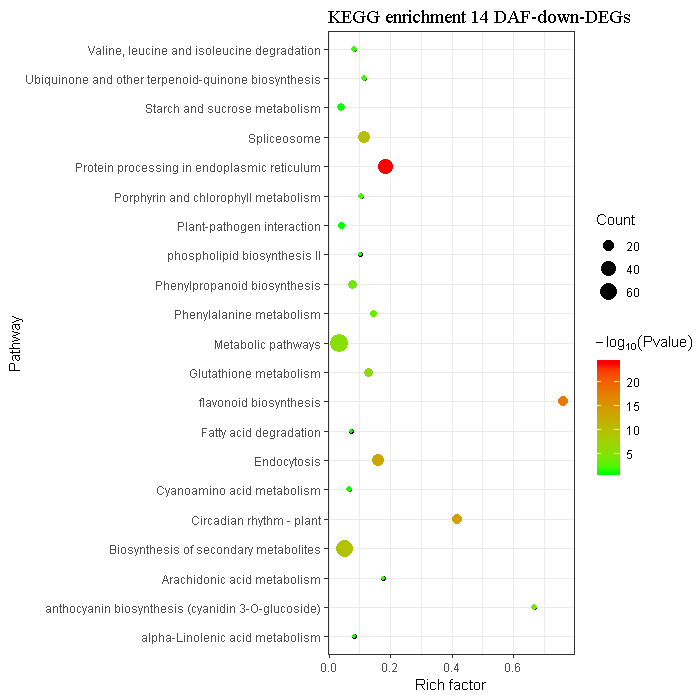


(b)


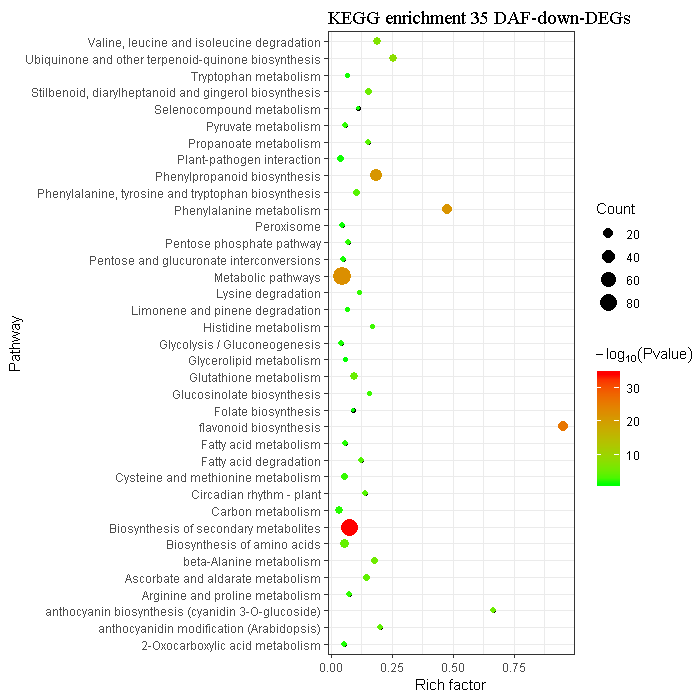


(c)


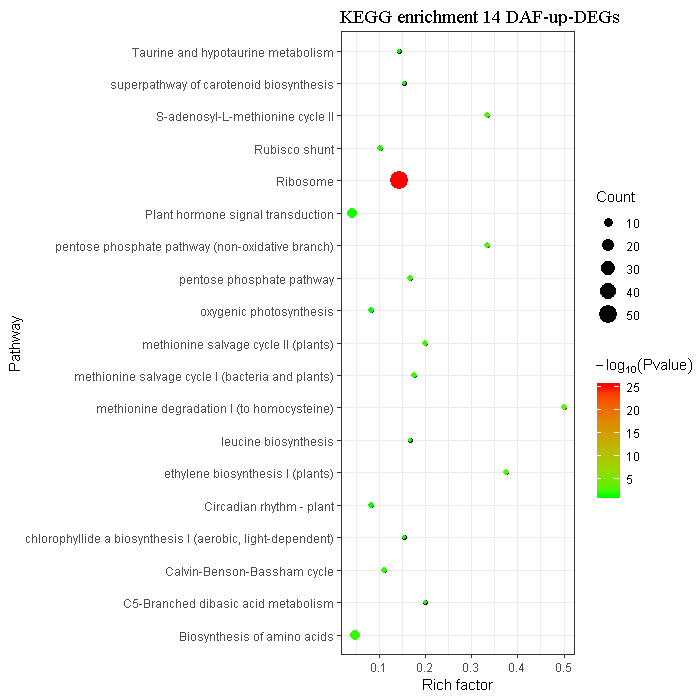


(d)


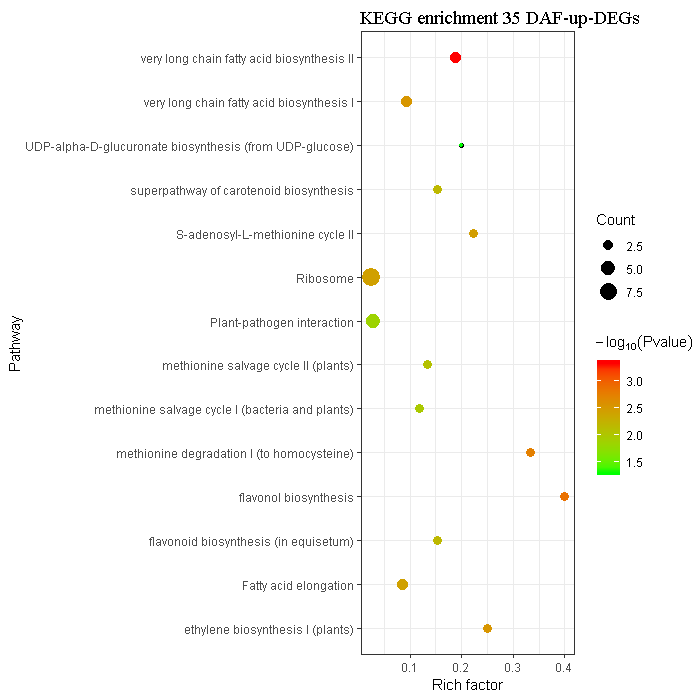


**Fig. S11 Results of KEGG pathway of all up- and down-regulated genes.** (a) KEGG pathway of down-DEGs in 14 DAF; (b) KEGG pathway of down-DEGs in 35 DAF; (c) KEGG pathway of up-DEGs in 14 DAF; (d) KEGG pathway of up-DEGs in 35 DAF.


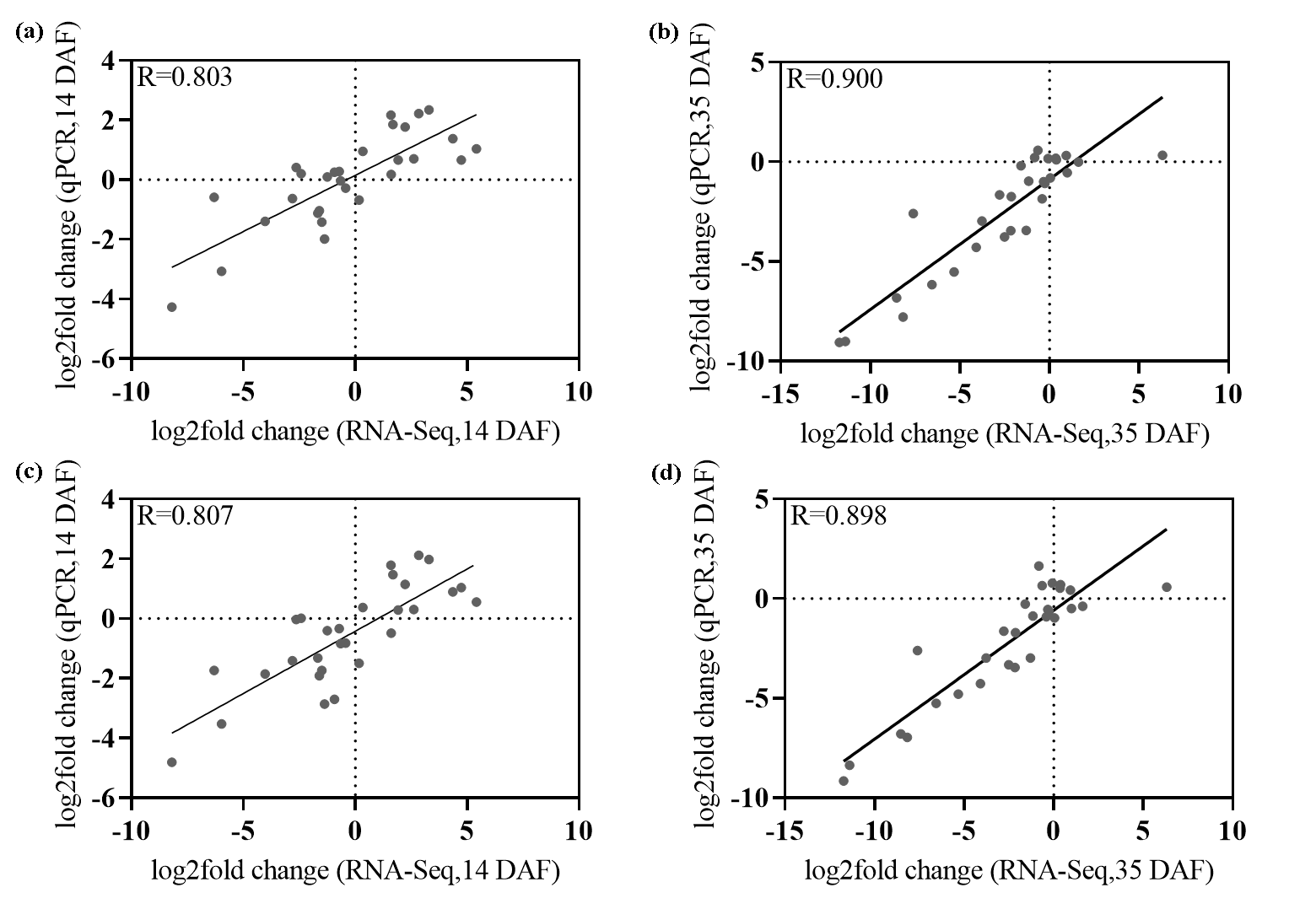


**Fig. S12 Validation of RNA-seq data by using qRT-PCR.** Expression abundance of 29 selected DEGs in seed coats at 14 DAF and 35 DAF is presented as the ratio of *BnTT8* double mutant (aacc) and WT. Data represent mean values ± SE of three biological replicates and three technical replicates of each sample. *B. napus* *PP2A-1* (a, b) and *ACT7* (c, d) genes were used as an internal control for data normalization. Correlation of the gene expression ratios between qRT-PCR and 14 DAF (a, c) or 35 DAF (b, d) RNA-seq data.
